# Fine-scaled climate variation in equatorial Africa revealed by modern and fossil primate teeth

**DOI:** 10.1073/pnas.2123366119

**Published:** 2022-08-22

**Authors:** Daniel R. Green, Janaina N. Ávila, Susanne Cote, Wendy Dirks, Daeun Lee, Christopher J. Poulsen, Ian S. Williams, Tanya M. Smith

**Affiliations:** ^a^Lamont-Doherty Earth Observatory and Climate School, Columbia University, New York, NY 10964;; ^b^Griffith Centre for Social and Cultural Research, Griffith University, Nathan, QLD 4111, Australia;; ^c^Department of Anthropology and Archaeology, University of Calgary, Calgary, AB T2N 1N4, Canada;; ^d^Department of Anthropology, Durham University, Durham DH1 3LE, United Kingdom;; ^e^Department of Earth and Environmental Sciences, University of Michigan, Ann Arbor, MI 48109;; ^f^Research School of Earth Sciences, Australian National University, Canberra, ACT 2601, Australia;; ^g^Australian Research Centre for Human Evolution, Griffith University, Nathan, QLD 4111, Australia

**Keywords:** primate ecology, Miocene apes, stable isotopes, seasonality, hominin evolution

## Abstract

Variability in resource availability is hypothesized to be a significant driver of primate adaptation and evolution, but most paleoclimate proxies cannot recover environmental seasonality on the scale of an individual lifespan. Oxygen isotope compositions (δ^18^O values) sampled at high spatial resolution in the dentitions of modern African primates (*n* = 2,352 near weekly measurements from 26 teeth) track concurrent seasonal precipitation, regional climatic patterns, discrete meteorological events, and niche partitioning. We leverage these data to contextualize the first δ^18^O values of two 17 Ma *Afropithecus turkanensis* individuals from Kalodirr, Kenya, from which we infer variably bimodal wet seasons, supported by rainfall reconstructions in a global Earth system model. *Afropithecus*’ δ^18^O fluctuations are intermediate in magnitude between those measured at high resolution in baboons (*Papio* spp.) living across a gradient of aridity and modern forest-dwelling chimpanzees (*Pan troglodytes verus*). This large-bodied Miocene ape consumed seasonally variable food and water sources enriched in ^18^O compared to contemporaneous terrestrial fauna (*n* = 66 fossil specimens). Reliance on fallback foods during documented dry seasons potentially contributed to novel dental features long considered adaptations to hard-object feeding. Developmentally informed microsampling recovers greater ecological complexity than conventional isotope sampling; the two Miocene apes (*n* = 248 near weekly measurements) evince as great a range of seasonal δ^18^O variation as more time-averaged bulk measurements from 101 eastern African Plio-Pleistocene hominins and 42 papionins spanning 4 million y. These results reveal unprecedented environmental histories in primate teeth and suggest a framework for evaluating climate change and primate paleoecology throughout the Cenozoic.

## Significance

Environmental variability may have spurred unique adaptations among Miocene apes and later hominins, but this hypothesis has been impossible to test on the scale relevant to individual lifespans. We establish that oxygen isotope compositions in modern primate teeth record annual and semiannual seasonal rainfall patterns across a broad range of environments in equatorial Africa. We then document annual dry seasons experienced by the large-bodied Early Miocene ape *Afropithecus turkanensis*, which may explain its novel dental adaptations and prolonged development. By revealing real-time historical and prehistoric environmental variation on a near weekly basis, we demonstrate that extraordinary behavioral and ecological variability can be recovered from modern and fossil African primates.

Climate seasonality is a potent driver of competition and natural selection (1), and is proposed to have influenced the origin and evolution of the great apes (2, 3), early hominins (4), and modern humans (5). Seasonal variation in water availability shapes the type and extent of savanna ecosystems (6), the expansion of which has exerted a profound effect on African faunal communities since the Late Miocene (7). In the Plio-Pleistocene, hominins occupied variably open environments (4, 8, 9), while African great apes were becoming largely confined to forests. Although long-term Neogene climate trends are reasonably well known, the ecologies of early apes are poorly understood. Detailed environmental records from the Early and Middle Miocene are scarce, but could clarify when and why the body plans, life histories, and foraging patterns of modern great apes evolved (3, 10). The large-bodied Early Miocene ape *Afropithecus turkanensis* is of particular interest in this regard. *Afropithecus* is the oldest-known ape to possess anterior tooth specializations for hard-object feeding and thick molar enamel, as well as the first to have grown its molars over an extended period similar to modern African apes, features widely considered to be behavioral and developmental adaptations to durophagy and seasonal fallback food reliance (2, 11–14). Seasonal dietary resources are believed to have been important for later Eurasian apes of the Middle Miocene, and remain an important part of modern ape ecology (2, 14, 15).

Innovations in isotopic sampling methods now permit investigation of the seasonal ecology of extinct primates. While most paleoenvironmental proxies aggregate processes in sediments over thousands of years or longer—obscuring consequential trends in seasonality—ephemeral climate variation can be inferred from sequential changes in the composition of fossil teeth. Oxygen isotope compositions exhibit fluctuations that are recorded as teeth grow over time because hydroxyapatite mineral forms in equilibrium with body water δ^18^O values (16, 17). In tropical climates relevant to the evolution of apes, wet seasons result in low δ^18^O values, whereas during dry seasons the δ^18^O values of meteoric, surface and leaf waters increase (18–20). These patterns are influenced by differences in moisture sources and surface evaporation, as well as by altitude, floral communities, and animals’ water tolerance, impacting mammalian δ^18^O values across Africa at regional and local scales (18–25). It is possible to recover elements of this hydrological, physiological, and behavioral variability at high temporal resolution by sampling the rapidly mineralizing innermost enamel adjacent to the enamel–dentine junction (EDJ). This location averages far less time than other regions of the enamel, and δ^18^O measurements can be coupled with daily incremental tooth formation to determine the timing of climatological and physiological events (17, 26–28). It remains unclear, however, to what extent primate enamel δ^18^O values reveal specific meteorological histories, or how these values reflect hydrology at regional and continental scales.

Here we first test whether primate enamel oxygen isotope compositions track meteorological histories in two baboons (*Papio hamadryas*) by contrasting enamel δ^18^O measurements with concurrent local rainfall records. We quantify daily tooth growth rates to facilitate near weekly, fine-scale (approximately 15 to 20 µm) sequential δ^18^O measurements over multiple years of formation using a sensitive high-resolution ion microprobe specialized for stable isotope analysis (SHRIMP-SI). Baboon individuals belonged to two troops relying upon different, nearby water sources—the shallow, saline Lake Basaka, and the Awash River, buffered by the upstream Koka Dam—permitting an examination of how local hydrology may mediate the incorporation of environmental signals into teeth. We then test whether enamel δ^18^O values from primates in five populations across equatorial Africa evince broader regional patterns of rainfall seasonality and isotopic compositions. Long-term environmental comparisons have established that, relative to the eastern African Rift region and highlands, densely forested areas of western Africa have more consistent and higher annual rainfall, and rainfall δ^18^O values are lower. In parts of eastern Africa, wet seasons are both annual and semiannual, and arid regions tend to experience variable and less rainfall overall; rainfall tends to have higher δ^18^O values due, in part, to ^18^O-enriched moisture sources and evaporative effects (18, 22).

Finally, to improve the record of Early Miocene climate relevant to the environments in which apes originated and evolved, we investigate the oxygen isotope ecology of *A. turkanensis* and fossil herbivores from the circa 17 Ma Kenyan site of Kalodirr (11, 29, 30). Our two *Afropithecus* specimens derive from different stratigraphic levels within Kalodirr’s depositional sequence, allowing a test of the stability of the environments *Afropithecus* occupied over a portion of the Kalodirr Member. These results are compared to enamel δ^18^O values from fauna sampled using traditional hand-drilled (bulk) carbonate methods, and contextualized with our analyses of modern African cercopithecoids and chimpanzees (Table 1). Values are also compared with simulations of seasonal Miocene rainfall and precipitation δ^18^O from an Earth system model (31). Earth system models are three-dimensional numerical models of the global climate system that resolve the circulation and climate features of the atmosphere, ocean, and land surface. They are best known for their use in making future climate projections in response to anthropogenic emissions. For this study, we have modified an Earth system model, the National Center for Atmospheric Research Community Earth System Model (31), to simulate past climate conditions by incorporating nonvarying boundary conditions (e.g., atmospheric CO_2_ levels, geography, bathymetry, surface elevations, surface types, glacial ice, aerosols, and solar luminosity) appropriate for the Miocene. The model simulates subdaily climate features at a horizonal resolution of 1.9° × 2.5° in the atmosphere and 1° × 1° in the ocean; however, in this study we focus on seasonal timescales. This integration of traditional and high spatial resolution stable isotope compositions, coupled with Earth system modeling, provides insights into the environmental conditions directly experienced by this important ancient ape.

**Table 1.** Sample of modern and fossil primate teeth in the present study

Taxon	Region	Individual ID/accession	Teeth	Years sampled
*Papio hamadryas* sp.	Awash National Park, Ethiopia	73261	LM1-3	4.7
*Papio hamadryas sp.*	Awash National Park, Ethiopia	73436	LM1-2	3.4
*Papio anubis* sp.	Awash National Park, Ethiopia	HT 17-02	LM2-3	3.3
*Papio anubis sp.*	Awash National Park, Ethiopia	HT 18-02	LM2-3	3.4
*Papio anubis-hamadryas* sp.	Awash National Park, Ethiopia	HT 19-02	LM2-3	2.5
*Theropithecus gelada*	Debre Highlands, Ethiopia	HKU 0237	LM2	2.2
*Theropithecus gelada*	Debre Highlands, Ethiopia	HKU 0243	LM3	2.9
*Papio anubis*	Bushenyi District, Uganda	U9	LC, LM1-2	7.1
*Papio anubis*	Bushenyi District, Uganda	U10	UC, UM1	2.2
*Chlorocebus tantalus tantalus*	Bushenyi District, Uganda	HT 06-02	LM1-3	4.0
*Chlorocebus tantalus tantalus*	Bushenyi District, Uganda	HT 07-02	LM1	1.0
*Cercopithecus mona*	Lama Forest, Benin	HT 01-10	LC, LM1	3.5
*Pan troglodytes verus*	Ganta Region, Liberia	7038	UM3	2.5
*Pan troglodytes verus*	Ganta Region, Liberia	7079	UM3	2.8
*Afropithecus turkanensis*	Kalodirr, Kenya	KNM-WK 17024	LM2	2.7
*Afropithecus turkanensis*	Kalodirr, Kenya	KNM-WK 24300	LM2	3.2

## Results

### Eastern African Modern Primates.

**Fig. 1.**
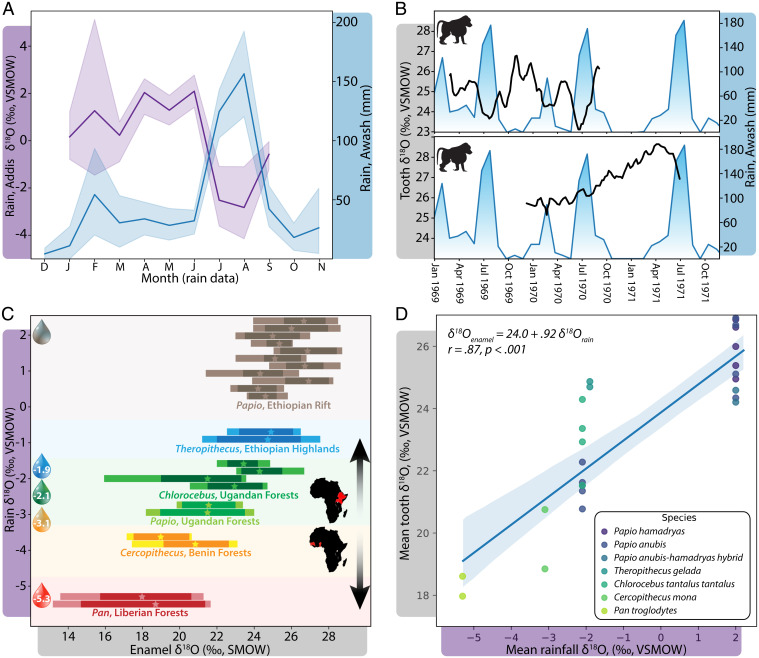
Primate enamel δ^18^O values reflect local seasonal rainfall and regional hydrology. (*A*) Seasonal meteoric precipitation amounts from the Awash National Park (blue trendline) are reflected by rainfall δ^18^O compositions from Addis Ababa (purple trendline) averaged over 1966 to 1973. Measured trends in water near the headlands of the Awash River are largely consistent with rainfall amounts in the national park over the lifetime of baboons studied here. (*B*) Three-sample moving average of weekly sequential enamel δ^18^O values (black trendline) from the M3 of 73261 (*Upper*) and M2 of 73436 (*Lower*), registered to contemporaneous local rainfall values over 1969 to 1971 (blue trendline). Error margins from the estimated duration of tooth development prior to death in 1973 precluded the calendar registration of earlier-forming molar teeth or more precise alignments of these teeth. (*C*) δ^18^O measurement ranges from cercopithecoid teeth (horizontal bars) at Awash in the Ethiopian Rift Valley (brown), Debre in the Ethiopian highlands (blue), Bushenyi District in Uganda (green), Lama Forest in Benin (yellow), and chimpanzee teeth from the Ganta region in Liberia (red). Means are shown as stars and 95th percentiles shown as darkened bar segments. On the *y* axis, mean estimated rainfall δ^18^O is shown for each of the five sites, demonstrating that primate enamel δ^18^O follows regional rainfall δ^18^O. Horizontal enamel δ^18^O bars are not scaled to the *y* axis. (*D*) Estimated mean annual rainfall δ^18^O (33–35) compared to enamel δ^18^O tooth means in this study.

We first establish an association between local monthly rainfall amounts, δ^18^O measurements from an upstream water source, and δ^18^O values from modern *P. hamadryas* tooth enamel formed concurrently. Primary monsoon rains arrive in Ethiopia’s Awash National Park in July and August, and are accompanied by a synchronous rapid decrease in rainwater δ^18^O values in the Ethiopian highlands (Fig. 1*A*) (32, 33). δ^18^O values were measured from several molars of two baboons that died in 1973; the formation of two of these molars during 1969 to 1971 facilitates comparison with measured local rainfall (Fig. 1 *A* and *B*, *SI Appendix*, *SI Text* 1.1 and Figs. S1 and S2, and Dataset S1). The third molar (M3) cusp of baboon 73261, whose troop drank from Lake Basaka, reveals multiple δ^18^O troughs with the same timing as the primary rains of 1969 and 1970. The second molar (M2) cusp of baboon 73436, whose troop lived next to the Awash River, shows a steady increase in δ^18^O values over more than 8 mo that is consistent with the marked drought from September 1970 until May 1971, a period during which the minor seasonal rain failed to arrive. Values for this M2 finally decline, presumably with the onset of the 1971 rains.

While δ^18^O measurements of additional molars from these two individuals and other nearby wild baboons cannot be precisely anchored in time (and thus related to rainfall records), enamel δ^18^O means and ranges from a total of five individuals from Awash National Park are relatively consistent (Fig. 1*C* and *SI Appendix*, Figs. S1–S5). Their range of variation (on average 3.6‰) is slightly less than average seasonal rainfall δ^18^O variation of 4.9‰ in the Addis highlands, upstream from the Awash River (Fig. 1*A*). Major peaks and troughs in the enamel δ^18^O of Awash baboons show annual periodicities across most but not all molars (*SI Appendix*, Figs. S6–S10), while some teeth also record the semiannual rainfall periodicities present in this region of eastern Africa (22, 33).

When looking more broadly across eastern Africa, enamel δ^18^O values are highest in *P. hamadryas* baboons inhabiting arid scrubland where estimated rainfall δ^18^O values are also high (33, 34). Mean values are lower in teeth from two highland *Theropithecus gelada* individuals (24.8‰, *n* = 235 measurements) (*SI Appendix*, *SI Text* 1.2 and Figs. S11 and S12 1.2), where annual rainfall is greater and rainfall δ^18^O values are correspondingly lower (inferred from nearby Addis and from regional rainfall δ^18^O models) (33–35). Enamel δ^18^O values are lowest in two Ugandan baboons (*Papio anubis*) from the Bushenyi District (21.8‰, *n* = 544 measurements) (*SI Appendix*, *SI Text* 1.3, and Figs. S13–S15), where rainfall mean δ^18^O values are also lowest. Two Bushenyi tantalus monkeys (*Chlorocebus tantalus*) have slightly elevated δ^18^O values (23.0‰, *n* = 267 measurements) (*SI Appendix*, Figs. S16–S18) compared to the contemporaneous sympatric baboons, consistent with their relatively high ingestion of arboreal resources that are likely enriched in ^18^O (19, 20, 25). A pronounced but brief decline in δ^18^O values in one tantalus monkey may correspond to a major rainstorm in April 1963 coincident with third molar formation (*SI Appendix*, Fig. S17); historical rainfall records document the end of a long drought at this time, and a 60-y low in rainfall δ^18^O measurements from nearby Entebbe (33).

### Western African Modern Primates.

Comparisons across equatorial Africa reveal that primate enamel δ^18^O values mirror regional hydroclimate and oxygen isotope systematics (Fig. 1 *C* and *D* and Datasets S1 and S2). Primate enamel δ^18^O values are highest in eastern Africa, where rainfall δ^18^O values are also high, and lower in western Africa (Fig. 1*C*) (33–35). Mean enamel δ^18^O measurements correlate relatively closely with mean annual rainfall δ^18^O estimates (Fig. 1*D*); while the relationship between variability in tooth and rainfall oxygen isotope composition is positive, it is inconsistent (*SI Appendix*, Fig. S19).

**Fig. 2.**
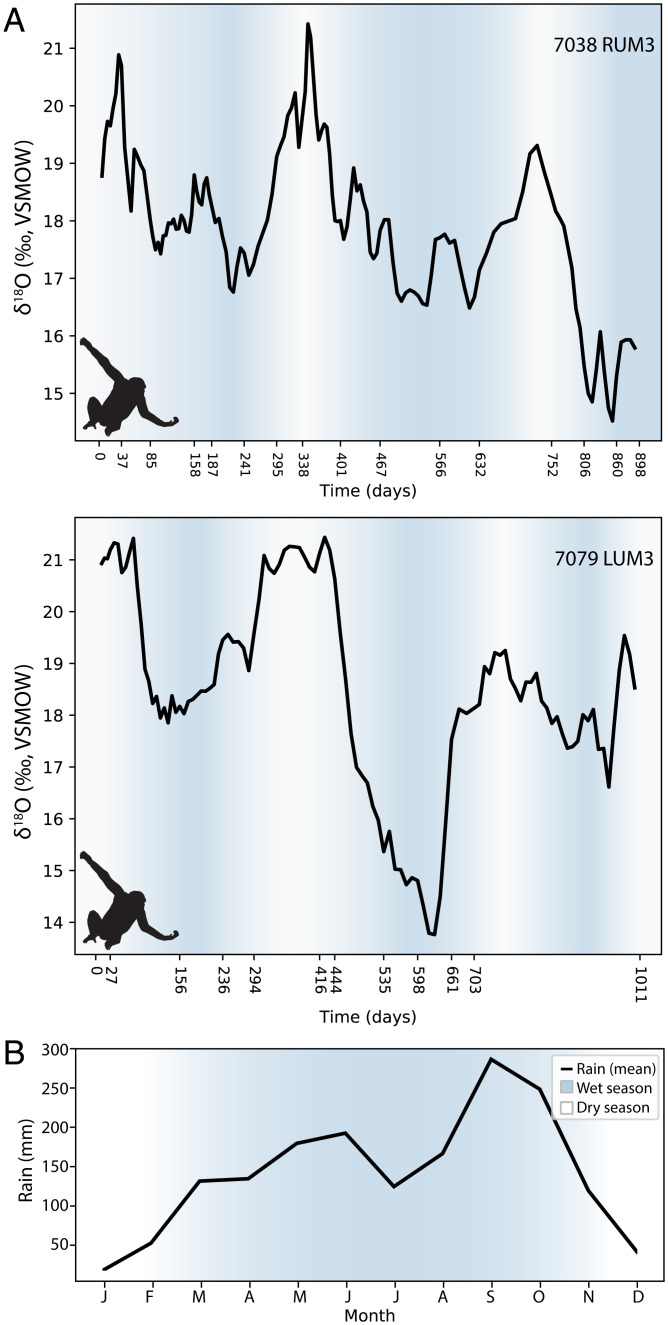
δ^18^O values from two Liberian chimpanzee (*Pan troglodytes verus*) molars reproduce expected annual wet and dry seasons. (*A*) Three-sample moving average of weekly δ^18^O values (in days) for individual 7038 (*Upper*) and 7079 (*Lower*); inferred wet seasons are shown in blue, while dry seasons are unshaded. (*B*) Mean annual rainfall in the Taï Forest (black line) is similar to that observed in nearby Liberia, where there is one annual highly consistent dry season (December to February), and a longer rainy season punctuated by a brief midseason reduction in rainfall.

Two M3s from broadly contemporaneous Liberian chimpanzees show mean δ^18^O values (18.3‰) lower than those of the Ethiopian rift baboons (25.6‰) and highland geladas (24.8‰), Ugandan baboons (21.6‰), and tantalus monkeys (23.0‰) (*SI Appendix*, *SI Text* 1.4). Similar seasonal patterns are seen in both chimpanzees, with δ^18^O ranges of 7.9‰ and 8.7‰, respectively (Fig. 2, *SI Appendix*, *SI Text* 1.5, and Dataset S2). The M3 of individual 7038 was sampled over 2.5 y of continuous growth, revealing three discrete δ^18^O peaks that are likely capturing brief dry seasons. The M3 of individual 7079 yielded 2.8 y of records, revealing three dry seasons more prolonged than those experienced by 7038. Frequency analyses show 1.0-y periodicities in δ^18^O values in the molars of both individuals (*SI Appendix*, Fig. S20). Low enamel δ^18^O values and strong annual oscillations are consistent with records of high rainfall (approximately 1,880 mm annually) at the time of collection in the Ganta region of Liberia (36), similar to recent patterns from the Taï Forest, Ivory Coast (Fig. 2*B*) (37).

We also sampled one mona monkey (*Cercopithecus mona*) from Benin, where chimpanzees are now locally extinct. Its higher mean enamel δ^18^O value (19.8‰, *n* = 181 measurements) (*SI Appendix*, Fig. S21 and Dataset S1) is consistent with higher rainfall δ^18^O values in Benin compared to Ganta (33–35), and with feeding in a higher canopy niche relative to chimpanzees. Weekly δ^18^O measurements over 2.6 y of tooth formation reveal the same strongly annual seasonality as in the Liberian chimpanzees (*SI Appendix*, Fig. S22).

### *A. turkanensis* and Associated Fauna.

**Fig. 3.**
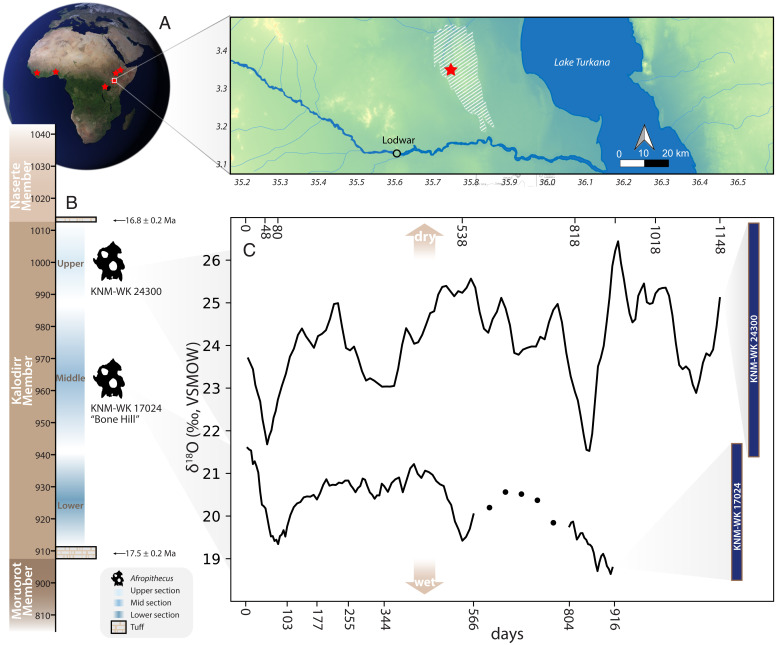
*Afropithecus* comparative, geographic, and stratigraphic context. (*A*) Primate samples (red stars) derive from Liberia (chimpanzees), Benin (mona monkey), Ethiopia (geladas and baboons), Uganda (tantalus monkeys and baboons), and Kenya (*Afropithecus)*, with the latter sampled from the Kalodirr member of the Lothidok Range (white hashes on *Inset* map). (*B*) Kalodirr stratigraphy spanning 17.5 to 16.8 Ma, with *Afropithecus* specimens found at middle and upper portions of the sequence. (*C*) A three-sample moving average of weekly δ^18^O values (in days) for *Afropithecus* individual KNM-WK 24300 (*Upper*) and KNM-WK 17024 (*Lower*). Dots show measurements in a region of modified enamel of KNM-WK 17024 that precluded fine-scaled continuous sampling. Full δ^18^O ranges for each individual are shown in dark blue on the right. Drier conditions experienced by *Afropithecus* are associated with higher δ^18^O values above, and wetter conditions by lower values below (brown arrows).

**Fig. 4.**
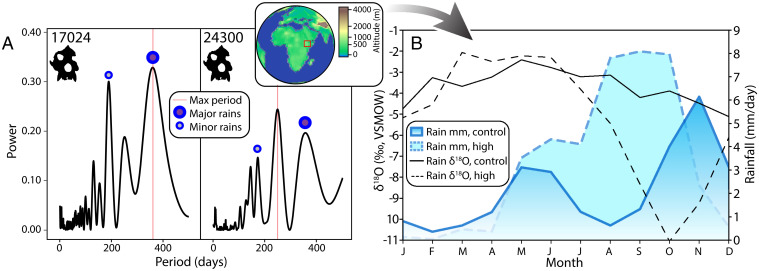
Frequency analysis and climate modeling indicate annual and semiannual seasonality in Early Miocene eastern Africa. (*A*) Lomb–Scargle periodograms evaluating δ^18^O records from *Afropithecus* teeth show that approximately 6-mo (“minor rains,” small blue circle) and 12-mo (“major rains,” large blue circle) periods, and an additional subannual period in KNM-WK 24300, account for most of the temporal patterns. The strongest period is marked by a red line. (*B*) Global earth system model for the eastern African Miocene fully coupled to global atmospheric and ocean circulation patterns predicts semiannual rainfall patterns under control conditions (dark blue) and annual rainfall patterns high insolation conditions (light blue) in the Turkana region around Kalodirr; annual seasonality in rainfall δ^18^O is simulated under control (solid black line) and high insolation (dashed line) orbital configuration. *Inset* map shows predicted Miocene elevation contributing to model results.

*Afropithecus* specimens KNM-WK 17024 and KNM-WK 24300 were collected from the middle and upper layers of the Kalodirr Member of the Lothidok Formation, respectively (Fig. 3 *A* and *B*). Specimen KNM-WK 17024 derives from a fossiliferous locality in the middle of the Kalodirr Member known as “Bone Hill,” whose precise age is unknown but is bounded by the tuff at the base of the member (17.5 ± 0.2 Ma) and the Naserte Tuff above (16.8 ± 0.2 Ma) (Fig. 3*B*) (30). KNM-WK 24300 was recovered from sediments immediately below the Naserte Tuff, and we therefore estimate the age to be slightly older than 16.8 Ma. Fine-scaled enamel δ^18^O measurements show ranges of 3.3‰ (KNM-WK 17024, *n* = 113) and 5.5‰ (KNM-WK 24300, *n* = 135), or 8.4‰ across both teeth combined (Fig. 3*C*, *SI Appendix*, *SI Text* 1.6, and Dataset S3). The higher δ^18^O values and larger δ^18^O range in KNM-WK 24300 (21.4 to 26.9‰) compared to KNM-WK 17024 (18.5 to 21.8‰) suggest a landscape for the individual in the upper layer that was more seasonally variable, and possibly drier, which is discussed further below. KNM-WK 24300 appears to have experienced three prolonged dry seasons over 3 y, with the second lasting 7 to 9 mo. The M2 of individual KNM-WK 17024 appears to show two sustained dry periods over 2.5 y. The strongest seasonal oscillations are annual in KNM-WK 17024 (1.0 y) and subannual in KNM-WK 24300 (0.7 y), although KNM-WK 24300 also records a sharp secondary peak at 1.0 y (Fig. 4*A*).

Enamel carbonate δ^18^O values from Kalodirr terrestrial herbivores (*n* = 66 individuals) (*SI Appendix*, *SI Text* 1.7 and 1.8 and Dataset S4; see *Materials and Methods* for carbonate-bioapatite comparison) demonstrate that anthracotheres (*n* = 8) have the lowest δ^18^O values (mean 17.9‰, Vienna Standard Mean Ocean Water, VSMOW), consistent with an aquatic or semiaquatic ecology (38). In contrast, suids (*n* = 7), hyraxes (*n* = 7), rhinocerotids (*n* = 7), and giraffids (*n* = 5) have higher δ^18^O values (means 19.7 to 19.8‰), consistent with terrestrial browsing. Anthracotheres—taxa most likely to reflect site hydrology due to inferred semiaquatic behaviors—have elevated δ^18^O values in the upper portion of the sequence, suggesting a hydrological shift, and possibly drier conditions in younger deposits. This would be consistent with the elevated δ^18^O values observed in the younger *Afropithecus* specimen (KNM-WK 24300). Mean δ^18^O values from both *Afropithecus* individuals are elevated by 3.2‰ compared to contemporary Kalodirr terrestrial herbivores (*SI Appendix*, Fig. S23). This difference is similar to that observed between chimpanzees and terrestrial herbivores at Kibale, Uganda (2.2‰) (*SI Appendix*, Fig. S24) (25), supporting the fidelity of niche reconstruction from fossil enamel δ^18^O values at Kalodirr.

To further evaluate seasonal δ^18^O oscillations observed in *Afropithecus* molars, we deploy a water isotope-enabled Earth system model (31) to predict seasonal rainfall patterns on a similarly detailed scale. The model fully resolves atmosphere, ocean, and land surface processes, and is framed by Miocene paleogeography, bathymetry, atmospheric CO_2_, and ice volumes (*SI Appendix*, *SI Text* 1.9). Due to the significant influence of Earth’s orbital configuration on low-latitude precipitation, we ran simulations under two different orbital configurations, a “control” run with modern orbital parameters and a “high insolation” run with orbital parameters that maximize Northern Hemisphere summer insolation. In the control, the model predicts semiannual rains at Kalodirr 17 Ma, consistent with the high-resolution tooth δ^18^O measurements and periodograms from *Afropithecus* individual KNM-WK 24300 (Fig. 4). In the high insolation run, the model predicts a continuous wet season from May to November with greater maximum rainfall, resembling the more annual pattern observed in individual KNM-WK 17024. Under control conditions, simulated precipitation δ^18^O values at Kalodirr have a seasonal range of approximately 2.7‰ with a maximum during April to June and a minimum in November to January; this range increases to 9.0‰ with high insolation.

## Discussion

### Primate Enamel δ^18^O Values Track Equatorial African Hydrology.

We have established that oxygen isotope compositions in primate teeth reflect environmental gradients in rainfall amount, altitude, and aridity. Enamel δ^18^O values are highest in the arid Ethiopian rift, intermediate in Uganda, and lowest near the Guinean coast, mirroring continental-scale rainfall δ^18^O patterns (Fig. 1) (15, 33–35). Comparisons of primate populations in the same region show that local topography impacts δ^18^O values, as expected given rainout of air masses over rising altitudes (15, 20); highland gelada values are lower than lowland rift baboons. When populations are compared from the same location near Bushenyi District, Uganda, we find that enamel δ^18^O values segregate with ecological niches. Higher δ^18^O values among tantalus monkeys relative to local baboons (Fig. 2*C*) suggest the preferential consumption of arboreal resources, as is the case with other African primates feeding at different canopy heights (19, 23–25).

In addition to reflecting broad environmental trends, primate oxygen isotope compositions reveal specific meteorological histories and human alteration of the hydrological landscape. Awash baboon values are largely consistent with contemporaneous records of steep gradients in local rainfall and the δ^18^O values of precipitation in the upstream highlands (Fig. 1 *A* and *B*). Within the Awash individuals, enamel δ^18^O values differ between one baboon that drank from the shallow mineral Lake Basaka and another with year-round access to the Awash River. The latter individual’s isotopic compositions were likely buffered by the operation of the Koka Dam and reservoir upriver, with a total volume approximately five times that of Lake Basaka. Differences in the enamel δ^18^O profiles of two baboons experiencing similar rainfall histories, but living adjacent to different water bodies, suggests that local hydrology is a potent mediator between rainfall and body water isotope compositions. These differences also reveal how human alterations of the hydrological landscape can influence the body chemistry of local wildlife.

Our data show seasonal patterns consistent with trends in western and central Africa; enamel δ^18^O measurements in the chimpanzees and mona monkey reveal annual wet seasons, consistent with dominant rainfall patterns in this region (33–37). Rainfall seasonality, rainfall δ^18^O variation, and local variability in river water δ^18^O are estimated to be high in portions of the Gulf of Guinea (18, 33–35), likely contributing to the large enamel δ^18^O ranges in Liberian chimpanzees. Variable drinking and foraging behaviors may further contribute to enamel δ^18^O variability: across Africa, chimpanzees acquire water from rivers, streams, tree hollows, soaked wood, and underground tubers (39). Chimpanzees in the nearby Taï Forest consume at least 263 plant species with strong seasonal preferences (40). Primate enamel δ^18^O values may serve as faithful proxies for local hydrology and environmental variation despite the complexity of feeding behaviors (21, 24, 25, 37, 39, 40), because primates rely on plant resources with isotopic compositions that are exquisitely sensitive to local temperature, humidity, and evapotranspiration (40, 41). In summary, primate enamel δ^18^O profiles capture local environmental variation and broad rainfall patterns across Africa, demonstrating their utility for climatic and hydrological reconstruction.

### Climatic Drivers of Miocene Ape Evolution.

The two *Afropithecus* specimens collected from different layers in the Kalodirr Member show enamel δ^18^O profiles consistent with semiannual and annual dry seasons of variable intensity. As in modern ecosystems where arboreal primate δ^18^O values are elevated relative to sympatric terrestrial herbivores, enamel δ^18^O values of *Afropithecus* are higher than other fossil taxa at Kalodirr (*SI Appendix*, Figs. S23 and S24), supporting reconstructions of their arboreal feeding ecology (2, 12, 13, 30). Sustained durations of high δ^18^O values suggest longer dry seasons for *Afropithecus* than those experienced by Liberian chimpanzees, and the semiannual wet seasons seen in the younger specimen (KNM-WK 24300) resemble rainfall patterns like those sampled from primate teeth in arid regions of the contemporary eastern African Rift (Fig. 4*A*).

Our Earth system model shows that Early Miocene rainfall at Kalodirr could be semiannual or annual depending on the orbital configuration, consistent with *Afropithecus* seasonal δ^18^O profiles. The lower rainfall δ^18^O range simulated by our model under “control” orbital conditions appears consistent with the measured δ^18^O range in KNM-WK 17024. Simulated higher amplitude rainfall δ^18^O variation under conditions of high Northern Hemisphere insolation suggests that orbitally-forced climate differences may explain the higher enamel δ^18^O variation in KNM-WK 24300 relative to KNM-WK 17024. Additional factors may also contribute to differences between *Afropithecus* individuals, including variation in aridity and vegetation over time, or in temperature and global ice volume, although ocean δ^18^O records during this period indicate relative stability preceding the Miocene Climatic Optimum (42). Behavior and site hydrology could also influence δ^18^O values, analogous to differences between baboons drinking from varied water sources in the Awash National Park (Fig. 1).

The environmental variation that we have reconstructed using δ^18^O values and Earth system modeling supports the suggestion that *Afropithecus*’ distinct tooth and jaw morphology allowed it to exploit seasonally variable and fallback resources (2, 14). Hard-object feeding (durophagy) in *Afropithecus* is well supported by facial musculature and skeletal buttressing, as well as a suite of dental characteristics including thick enamel (2, 11–14). For example, its canine placement and premolar size would have allowed for consumption of fruit protected by hard shells, nuts, or other mechanically demanding objects (11, 13). Characterizations of enamel thickness place *Afropithecus* at the high-end of Early Miocene and extant ape values (12), very similar to modern hard-object feeding mangabeys (43), though *Afropithecus*’ anterior dentition is morphologically distinct from mangabeys. Miocene apes may have also relied on cognitive adaptations for survival in challenging seasonal environments, differentiating them from cercopithecoids, which are thought to have responded to similar pressures with more rapid reproduction and anatomical specializations for folivory (3, 10). Social learning and extractive and cognitive behaviors needed to exploit seasonally variable and spatially complex resources likely required an extended developmental period (2, 3, 10–12, 15). Similar isotopic studies of Early and Middle Miocene apes *Ekembo*, *Proconsul*, *Equatorius*, and *Kenyapithecus* would provide important context about the relationship of these features to varied environments in eastern Africa, as well as heavily debated adaptions found in early hominins (2, 4, 8, 9).

### Implications for the Study of Primate and Hominin Paleoecology.

**Fig. 5.**
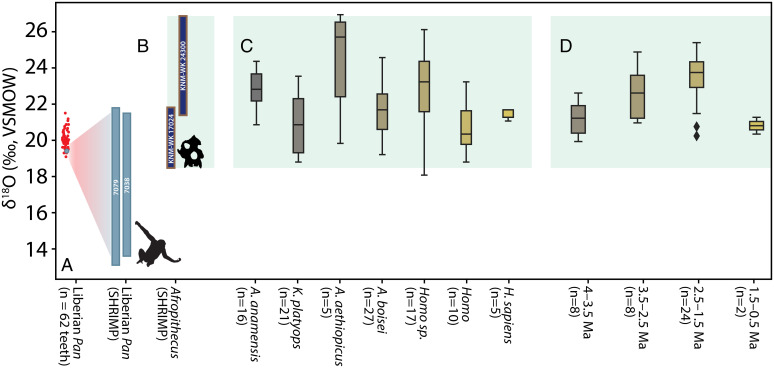
High-resolution δ^18^O samples from primate teeth reveal extensive lifetime climate variation relative to singular and aggregate bulk samples. (*A*) Conventional δ^18^O measurements from 62 teeth (red circles) of 33 chimpanzees reveals variation of only 2.7‰ (47). In contrast, high-resolution oxygen δ^18^O measurements of two chimpanzee molars (light blue bars) from the same population span 8.7‰. The gray circle is the single bulk measurement of chimpanzee 7079 (from ref. 47) converted from a carbonate measurement, as detailed in *SI Appendix*, *SI Text* 1.8. (*B*) δ^18^O ranges from two *Afropithecus* molars (dark blue) compared to (*C*) ranges derived from of bulk carbonate samples of 101 fossil hominins in the Turkana Basin over a 4-million-y period (45, 46) and (*D*) 42 bulk-sampled fossil *Theropithecus* individuals from an approximately 3-million-y period in the Turkana Basin (48). δ^18^O ranges of *Afropithecus* shown in *B*, shaded green across *C* and *D* for comparison.

Stable isotope measurements of drilled (bulk) enamel samples have contributed to the discovery of niche differentiation between fossil apes and hominins within their faunal communities (25, 44–46). Nevertheless, bulk stable isotope sampling may not reveal individual behavioral flexibility, nor population- or species-level seasonal variability, because bulk samples incorporate enamel formed over much longer periods of time than values from high spatial-resolution measurements (16, 17, 26–28, 45). We first illustrate this through the example of Liberian chimpanzees. Our high-resolution sampling of the M3s from two individuals (*n* = 255 measurements) reveals δ^18^O ranges (7.9‰ and 8.7‰) that are about three times larger than the range derived from bulk carbonate δ^18^O sampling of 33 individuals in this community (2.7‰, *n* = 62 teeth/measurements) (Fig. 5*A*) (47). A single bulk δ^18^O measurement of the M3 of chimpanzee 7079 yielded a phosphate-equivalent value of 19.4‰ (*SI Appendix*, *SI Text* 1.5 and 1.8) (47), while our 111 near weekly measurements from this same tooth produced a range of 13.1 to 21.8‰, with clear seasonal cycles over nearly 3 y (Fig. 2). Neanderthal teeth previously examined with this high-resolution approach also revealed a similar expansion in δ^18^O ranges relative to bulk samples, subsuming variation presumed to have distinguished different phases of local occupation (28).

Importantly, the two *Afropithecus* molars reveal isotopic variation that is nearly equal to bulk δ^18^O values from 101 Turkana Basin hominins spanning 4 million y (Fig. 5*C*) (43, 44). Similar δ^18^O measurements from 42 Turkana *Theropithecus* specimens also show a narrower range of variation than the *Afropithecus* molars (Fig. 5*D*) (48). Simply stated, bulk sampling of fossil primates (including hominins) is underrepresenting environmental variation and behavioral complexity. Microsampling oxygen isotopes in primate teeth compliments bulk approaches that explore population or species-level differences, and in this context can reveal crucial seasonal variation in the local hydrology and behavior of individuals. Future studies may elucidate details of past regional climates and even specific meteorological events, and will underpin the recovery of an extraordinary degree of ecological information from extinct taxa, including our primate ancestors.

## Materials and Methods

Primate samples are detailed in *SI Appendix*, *SI Text*. Thin-section production of *Afropithecus* is described in Smith et al. (49), chimpanzees in Smith et al. (50), and baboons in Dirks et al. (32); all formation times were revisited and updated here by T.M.S. following analytical procedures in Smith et al. (28). Daily growth lines were measured along enamel prisms from their initial formation over the dentine horn until an accentuated line was encountered, yielding the formation time of the corresponding segment of the EDJ. This process was then repeated while mapping the successive positions of the enamel growth front (expressed as accentuated lines) until the enamel cervix was reached, yielding a total time of molar cusp formation (*SI Appendix*, Figs. S26 and S27).

SHRIMP measurement methods also follow those detailed in Smith et al. (28). Briefly, oxygen isotope ratios were measured using the SHRIMP SI at the Australian National University. Glass-mounted polished sections of the *Afropithecus* fossils and modern primates (Table 1) were cleaned with petroleum spirit, RBS detergent solution, and Millipore water, dried for ≥24 h in a 60 °C vacuum oven, and coated with a thin (approximately 10 nm) layer of high-purity Al (and/or Au) before being placed in the SHRIMP SI under high vacuum for approximately 12 h prior to analysis by secondary ion mass spectrometry (SIMS). An approximately 1.5-nA, 15-kV beam of positive Cs ions was used to sequentially sputter a series of approximately 15- to 20-µm diameter spots in the innermost enamel adjacent to the EDJ from the dentine horn to the cervix. Negative O secondary ions were extracted at 10 kV, mass separated at approximately 3000R (M/ΔM) and measured in current mode using a multiple collector equipped with dual Faraday cups (resistors 10^11^ Ω for ^16^O, 10^12^ Ω for ^18^O). Charge on the sample surface was neutralized using a 1.2-kV focused electron beam. Each analysis consisted of 2-min preconditioning, during which electrometer baselines were measured, followed by optimization of the beam steering and 6 × 20-s measurements of ^18^O/^16^O ratios, giving a spot uncertainty of approximately 0.1‰ (1SE; ^16^O c. 1.5 GHz). Corrections for electron-induced secondary ion emission were made based on measurements before and after each analysis. δ^18^O values were calculated relative to mineral apatite standard Durango3 (9.8‰, VSMOW) that was measured repeatedly over the course of the ∼14 to 24-h period of data collection for each tooth (2 SD, approximately 0.5‰). Measurement spots were spaced as far apart as 300 µm near the dentine horn, and reduced to 25 to 30 µm apart toward the cervix, with spacing calculated to maintain near weekly sampling across all teeth.

In order to assess time-dependent patterns of tooth δ^18^O measurements, we used a frequency analysis algorithm known as the Lomb–Scargle periodogram (51). The Lomb–Scargle algorithm takes measurements that have been sampled unevenly or irregularly over a given interval, and estimates the power of sine wave periods within a given range to produce the temporal patterns present within those measurements. The method estimates periodicities that underlie more complex signals in a manner similar to Fourier transformations. For each tooth we calculated a fourth-order polynomial function predicting the timing of formation in days from the distance in mm along the tooth EDJ; this function was fitted using day of formation and EDJ length data (Datasets S1–S3). The function was then used to convert distances from SHRIMP measurements into estimates of days of tooth formation. Paired δ^18^O measurements and day of formation estimates were provided to the Lomb–Scargle periodogram algorithm hosted by the AstroPy 4.0.1 library run with Python 3.1. For individuals with teeth that formed over 600 d or more, periods from 0 to 500 d were analyzed for each tooth, and the maximum power of each period was reported in years.

Kalodirr herbivore teeth were placed in stratigraphic sequence using GPS locations on the basis of work by Boschetto et al. (52). Only specimens showing no or little visible diagenetic alteration were selected for isotopic analysis. Superficial enamel was removed and discarded by rotary drill, and then enamel powder samples were collected from the clean surface. Samples were not pretreated prior to analysis. Carbonate δ^18^O values were measured by Jason Curtis at the University of Florida, Gainesville, using a Finnigan-MAT 252 IRMS coupled with a Kiel III carbonate preparation device. Approximately 600 µg per analysis of sample and 30 to 50 µg of NBS-19 standard were reacted with purified phosphoric acid for 470 s to release CO_2_ for isotopic measurement. Carbonate δ^18^O V-PDB values were converted to bioapatite-equivalent values by subtracting 8‰ following the procedure outlined in the *SI Appendix*, *SI Text* 1.8, and then placed on a VSMOW scale; original and transformed data are given in Dataset S4.

We performed paleoclimate simulations using the fully coupled water isotope-enabled Community Earth System Model by the National Center for Atmospheric Research (31), with Miocene boundary conditions (e.g., atmospheric CO_2_ levels, geography, bathymetry, surface elevations, surface types, glacial ice, aerosols, and solar luminosity). Further details can be found in *SI Appendix*, *SI Text* 1.9.

## Supplementary Material

Supplementary File

Supplementary File

## Data Availability

All study data are included in the main text and supporting information.
