# Supplementary material for: Fine-scaled climate variation in equatorial Africa revealed by modern and fossil primate teeth
Source: Proc Natl Acad Sci U S A. 2022 Aug 22;119(35):e2123366119. doi: 10.1073/pnas.2123366119 (PMC9440354; doi:10.1073/pnas.2123366119)
Supplement: Supplementary File [file pnas.2123366119.sapp.pdf]

Supplementary Information for

Fine-Scaled Climate Variation in Equatorial Africa Revealed by Modern and Fossil Primate Teeth

Daniel R. Green[1*], Janaina N. Ávila[2], Susanne Cote[3], Wendy Dirks[4], Daeun Lee[5], Christopher J. Poulsen[5], Ian S. Williams[6], Tanya M. Smith[2,7]

[1]Lamont-Doherty Earth Observatory and Earth Institute, Columbia University
[2]Griffith Centre for Social and Cultural Research, Griffith University
[3]Department of Anthropology and Archaeology, University of Calgary
[4]Department of Anthropology, Durham University
[5]Department of Earth and Environmental Sciences, University of Michigan
[6]Research School of Earth Sciences, Australian National University
[7]Australian Research Centre for Human Evolution, Griffith University

Daniel R. Green
Email: dgreen@ldeo.columbia.edu

This PDF file includes:

Supplementary Information Text
1.1 Modern primate samples: Awash National Park baboons
1.2 Modern primate samples: Debre highland geladas
1.3 Modern primate samples: Bushenyi District baboons and tantalus monkeys
1.4 Modern primate samples: Lama Forest Reserve mona monkey
1.5 Modern primate samples: Ganta chimpanzees
1.6 Kalodirr fossils: *Afropithecus turkanensis*
1.7 Kalodirr fossils: Herbivores
1.8 Carbonate-bioapatite conversion
1.9 Global Climate Model (GCM) for the Turkana Miocene

Figures S1 to S27
SI References

Other supplementary materials for this manuscript include the following:

Datasets S1 to S4 (Excel spreadsheet)

1.1 Modern primate samples: Awash National Park baboons

Two baboons derive from the Awash National Park, a semiarid woodland and thornscrub landscape adjacent to the Awash river, at 1000 meters of elevation in the Ethiopian rift valley *(1)*. Dental specimens from 73261 (M_1-M_3) and from 73436 (M_1-M_2) are part of the Newcastle Collection on loan to Durham University (ultimately to be curated at the University of Kent), and derive from two separate groups, A and J, respectively, in a larger regional baboon population. Individual 73261 was estimated to have died at ~8 to 9 years of age, and 73436 at 6.3 to 6.5 years of age *(2)*.

Group A comprised two separate hamadryas groups treated as one during trapping operations in 1973 (*3)* with a few anubis members, some distance from the Awash River in a setting that included *Acacia tortilis* and thornscrub *(4)*. These individuals accessed water from sources other than the Awash, including Basaka, a shallow mineral lake *(1)*, and probably seasonal puddles during the rainy season *(5)*. Individual 73261 died on April 15, 1973, shortly after capture, when its M3 roots were still forming.

Group J (Nagel *(5)*'s group C3) by contrast lived in the canyon below the Awash falls along the river, providing a year round source of water, along with seasonal puddles and watercourses. The Awash is dammed upstream at Koka, producing a reservoir lake approximate 4–5 times the area and volume of Basaka and likely dampening water turnover and seasonal $\delta^{18}O$ by mixing. The canyon includes gallery forest along the river and thornscrub on the sides of the canyon below the group's sleeping cliffs.

Individual 73436 died on July 2, 1973, shortly after capture, when its M3 crowns were still forming. Microsatellites indicate that this individual contained some anubis ancestry *(4)*. Awash baboons rely upon a diet of fruits, seeds, shrubs, flowers, grass roots, corms from sedges, and some vertebrates and invertebrates. A variety of acacias are important to their diet, with different parts of the tree utilized in different seasons providing an important fallback food during the dry season *(1, 5)*. Though Group J was close to the Awash River and Falls, and Group A was some distance from the river at a mineral lake, hair $\delta^{18}O$ analyses from both groups show little difference *(1)*. Irregular growth rates and growth intervals in hair *(6)* may account for differences in baboon hair and tooth $\delta^{18}O$ measurements; our results appear to suggest the importance of serial sampling in teeth to reveal differences in hydrological environments between populations.

Individuals HT17-02, HT 18-02, and HT 19-02 were males likely shot during pest control operations at the margins of Awash National Park, near the Awash Falls, around the same time as individuals 73261 and 73436 were trapped. HT 17-02 and HT 18-02 were *Papio anubis*; morphologically, HT 19-02 appears to have some anubis-hamadryas characteristics and may be a hybrid specimen. Their exact dates of death are not known and their molars cannot be related to specific rainfall histories.

1.2 Modern primate samples: Debre highland geladas

Theropithecus gelada individuals HKU 0237 and HKU 0243 were collected by Frederick Peter Lisowski in 1966, west of Debre Berhan in the Ethiopian highlands (*7*). One hundred kilometers northeast of Addis Ababa, the Debre Berhan region includes altitudes spanning 1800 – 3500 meters, and rainfall is characterized by bimodal monsoons and mean annual precipitation of 400-700 mm *(8)*. HKU 0237 was a young male gelada and HKU 0243 a young female; after collection both specimens were curated by Lisowski at Hong Kong University *(7)*. Geladas in the Debre Berhan area live in a primarily treeless environment and may consume invertebrates, small mammals, roots, stems, flowers, fruit, or seeds, but they specialize in the consumption of grass leaves. Their diets include more crude fiber than other cercopithecoids, but they obtain a greater portion of nutrients from grass than other monkeys by carefully picking young or green blades using their fingers, including during dry seasons *(9)*.

1.3 Modern primate samples: Bushenyi District forest baboons and tantalus monkeys

Anubis or olive baboons (*Papio anubis*) U9 and U10 and tantalus monkeys (*Chlorocebus tantalus tantalus*) HT 06-02 and HT 07-02 were collected in 1965 during pest control operations. They were shot as members of crop raiding groups near Rubirizi, in what is now the Bushenyi district in south west Uganda, close to Queen Elizabeth National Park. Individual U9 was estimated to have died at 4.6 to 5.0 years of age, and U10 at 2.4 to 2.6 years of age *(2)*. Unpublished histological work estimated age at death around 4 years old for HT 06-02, and 1.3 years for HT 07-02.

The ecological conditions and habitat in the mid-1960s were described in detail by Rowell *(11)* in her study of baboons at the Ishasha River at the southern edge of the park. The Bushenyi district includes a part of the park in the middle of its western edge. Rowell emphasizes that human activity, including yearly burning, had a profound effect on the vegetation both outside and within the park at that time *(12)*.

The general habitat was one of grassland with gallery forest along rivers, including her study area. For the baboons and tantalus monkeys sampled here, this would have included agricultural activity, and crop raiding on maize and cassava *(12)*. Baboon diets included the roots, shoots, buds and fruits of the shrub *Maera edulis*, figs (e.g., *Ficus gualocarpa* <u>and</u> *Ficus barteri*), acacias (*Acacia sieberiana*) as well as a wide variety of seasonally available fruits, seeds, roots, leaves, and insects. The park is close to the equator with low seasonal variation. The temperature ranges from 32 C during the day to 19 ºC at night *(11)*.

Rowell describes a bimodal pattern of annual rainfall with two wet seasons centered around the equinoxes, will rainfalls all year *(11)*. She notes that it was unusual to have more than two weeks without rain, and that rainfall variation between one year to the next was as high as between the wet and dry seasons.

Tantalus monkeys are members of the *Chlorocebus* group, often collectively referred to as "vervets," and now known collectively as savanna monkeys *(13)*. Tantalus monkeys in Nigeria are primarily frugivorous with a diet composed of 49.2% fruit, 25.1% insects, 20.5% leaves, and 5.3% flowers *(14)*. There is undoubtedly overlap with baboon diets, as 38.5% of the tantalus monkey diet came from *Ficus* fruits and leaves, the fruit of which was also consumed by baboons *(11)*. Unlike the much larger baboons, they are not reported to eat underground food sources, such as tubers and roots. In Nigeria, tantalus monkeys foraged along the forest edge 72% of the time, 20% in forest fragments, and only 8% in grassland. Given the availability of these habitat types in Uganda's Queen Elizabeth National Park *(11)*, it seems likely that our sample would have had a similar diet to those in Nigeria, supplemented by crop raiding, as seen in U9 and U10, with overlapping but different ecological niches.

Although we do not know the specific water supply for these animals, baboons are obligate drinker *(1)* and studies of vervets (*C. pygerythrus*) have shown daily drinking, with increases in mortality when no standing water is available *(15, 16)*. According to modern maps, Rubirizi itself is in the Rubirizi District and is surrounded by small crater lakes, while the Bushenyi District is south of it with fewer lakes. Between the Bushenyi District and Rubirizi, there are several small lakes connected by streams. Given the preference of both baboons and *Chlorocebus* for gallery forest when available, and the continuous rainfall, it seems likely that these streams would provide drinking water year-round.

1.4 Modern primate samples: Lama Forest Reserve mona monkey

Cercopithecus mona has a broad distribution in diverse, forested regions along the eastern Guinean coast of western Africa. Individual HT 01-10 was collected by Reiko Goodwin in Lama Forest Reserve in southern Benin *(17)*, sometime between 1995-1997. The forest is situated between the Kouffo and Ouémé rivers, and is characterized by high annual rainfall (c. 1100 mm / year) and semiannual seasonality. Long, major wet seasons are typically accompanied by

inundation of the forest, while shorter dry seasons may occasionally include fires; rain typically continues but is reduced during secondary minor dry seasons. *C. mona* monkeys at Lama prefer ripe fruit, but consume unripe fruit or seeds seasonally when preferred fruit is unavailable. Mona monkeys may raid crops from local Holi farmers, who also hunt them *(17)*.

1.5 Modern primate samples: Ganta chimpanzees

Chimpanzee specimens (7038 upper right M3; 7079 upper left M3) were originally collected in the vicinity of the Ganta Mission in northern Liberia in the 1940s *(18)* and are curated by Harvard University's Peabody Museum of Archaeology and Ethnology. The area from which chimpanzees were hunted spanned between the St. Paul, St. John and Cestos rivers, abutted the borders of Guinea and the Côte D'Ivoire, and included swamps, forests and hills between 425 and 550 meters elevation above sea level. Rainfall was recorded for a decade by George Harley prior to the collection of chimpanzee specimens, and was characterized by a prolonged wet season extending from March until November, followed by a short dry season in the winter *(18, 19)*.

Meteoric and surface water $\delta^{18}O$ measurements are scarce in this region of Africa, with the exception of a few locations in Mali and Niger on the outskirts of the western Sahara *(20)*. Projected rainfall $\delta^{18}O$ compositions from the Online Isotopes in Precipitation Calculator (OIPC3.1), estimated to vary from +5.7 ‰ in February to -6.1 ‰ in August, may be biased by regional undersampling *(20-24*). The annual average coastal rainfall $\delta^{18}O$ values estimated from Benin are -3.1 ‰, with 1 S. D. of $\pm$ 1.4 ‰ when monthly variation is accounted for *(20-24)*, and rain and surface water $\delta^{18}O$ variation may be high along the Guinean coast *(25, 26)*. One specimen sampled in this study, 7079, was previously bulk sampled for carbonate by ref *(18)*. We derive a mean value of 18.6 ‰ (V-SMOW), while ref *(18)* measured -3.1 ‰ (V-PDB), or 18.4–19.4 ‰ (V-SMOW) after correcting for carbonate-bulk apatite compositional differences (see section 1.8 below). This difference of at most 0.8 ‰ is within the ~ 1 ‰ margin of error for carbonate to bulk apatite $\delta^{18}O$ conversion *(27, 28)*, and would not be surprising given the 8.7 ‰ variability measured within the tooth in our study.

1.6 Kalodirr fossils: *Afropithecus turkanensis*

The Kalodirr Site Complex is a large paleontological site that consists of several geographically distinct areas, all with exposures of the Kalodirr Member of the Lower Lothidok Formation *(29)*. KNM-WT 24300 was originally designated with field number 90 WT 1110, whose location is marked by aerial photos provided by Meave and Louise Leakey. Because the specimen is high in the section, likely just below the Naserte Tuffs, we assign it a tentative age of 16.8 +/- 0.2 Ma. KNM-WT 17024 was given field number 85 WT 654, a fossil locality at Kalodirr informally called "Bone Hill." The locality is also marked on aerial photos. Being in the middle of the stratigraphic section and Kalodirr Member, it is difficult to date precisely, but it lies between two dated beds of 16.8 +/- 0.2 Ma and 17.5 +/- 0.2 Ma. We tentatively estimate that KNM-WT 17024 is ~ 300,000 years older than KNM-WT 24300, but note that it might be as much as 500,000 years older, given uncertain sedimentation rates.

1.7 Kalodirr fossils: Herbivores

All fossil specimens from Kalodirr are housed in the National Museums of Kenya, Nairobi, Earth Sciences Department or the Turkana Basin Institute, Turkwel Station. Out of all 66 fossil herbivore specimens for which we have collected enamel carbonate oxygen isotope data, stratigraphic information is available for 33. The majority of specimens derive from the Kalodirr Main Site, but some were collected at the Leaf Site, also known as Kanukurinya *(29)*. While the sites are geographically distinct, there is stratigraphic overlap. Kanukurinya is located in the Kalodirr Tuffs at the base of the Kalodirr Member. Specimens from Kalodirr Main Site come from all parts of the Kalodirr Member and are assigned to lower, middle, mid-upper and upper positions (Dataset S4). Kanukurinya specimens are stratigraphically equivalent to low specimens

from Kalodirr Main Site. Specimens from the lower and Kanukurinya levels, and from the middle and mid-upper levels, are grouped together in Fig. S23. Specimens in the lower stratigraphic group derive from the Kalodirr Tuffs dated to 17.5 +/- 0.2 Ma, while specimens from the upper group were collected just below the Naserte Tuffs, 16.8 +/- 0.2 Ma. Kalodirr herbivore teeth were placed in stratigraphic sequence using GPS locations on the basis of work by ref *(29).*

It should be noted that when comparing modern fauna $\delta^{18}O$ values with those of fossil taxa from the past, modern $\delta^{18}O$ values will be slightly enriched as a result of higher global ice volume today than in the past, causing light isotope (^{16}O) sequestration at the poles. Both *Afropithecus* and other Kalodirr fauna will therefore have naturally lower $\delta^{18}O$ values based on lower global meteoric $\delta^{18}O$ during the early Miocene. This is expected to amount to a divergence of 1 ‰ or less from modern values *(30, 31)*. Given the nature of our analyses, we are mindful of this factor but do not adjust our measurements.

1.8 Carbonate-bioapatite conversion

Biological hydroxyapatite mineral (bioapatite) in enamel contains approximately 40 weight percent oxygen, which is primarily found in three molecular species: phosphate, hydroxyl, and carbonate; the latter substitutes for the other two species in the hydroxyapatite lattice *(32)*. Phosphate, the oxygen-bearing species most resistant to diagenetic alteration, contributes the vast majority (88 %) of oxygen to bioapatite, while the remaining oxygen is found in either carbonate (8 %), or hydroxyl ions (4 %), which are expected to exchange with other ions during the process of fossilization *(27, 28, 32)*.

Ablative sampling techniques including ion probe and laser ablation measure all sources of oxygen within bioapatite. Because most oxygen in bioapatite derives from phosphate, $\delta^{18}O$ measurements sampled through ablation are expected to be determined primarily by phosphate $\delta^{18}O$ compositions. Relative to phosphate oxygen, carbonate oxygen isotope compositions in bioapatite are higher by approximately 8–9 ‰, a factor determined by the temperature of precipitation for both species; hydroxyl by contrast is lower by approximately 16.6 ‰. The isotopic offsets of carbonates and hydroxyls relative to phosphates therefore counteract one another, and ablatively sampled $\delta^{18}O$ compositions in bioapatite closely resemble those of phosphate, with expected bioapatite-phosphate deviations on the order of 1 ‰ *(27, 28, 32)*. A high-resolution comparison of SHRIMP and phosphate $\delta^{18}O$ measurements from the enamel of an experimentally-raised sheep has demonstrated that SHRIMP and phosphate $\delta^{18}O$ compositions closely track one another, and both record experimentally delivered temporal variations in the $\delta^{18}O$ composition of water *(28)*. Here, we observe that 111 SHRIMP measurements from the M3 of chimpanzee 7079 results in a mean $\delta^{18}O$ value of 18.6 ‰ on the VSMOW scale. Carbonate $\delta^{18}O$ measurement from chimpanzee tooth 7079 *(18)* produced a $\delta^{18}O$ value of -3.1 ‰ on the VPDB scale, which when transformed onto the VSMOW scale results in a $\delta^{18}O$ value of 18.4–19.4 ‰ depending upon what $\delta^{18}O$ offset is expected for carbonate and phosphate. This example suggests that SHRIMP $\delta^{18}O$ measurements appear to match carbonate measurements.

Based on the above considerations, we adopt a SHRIMP-derived bioapatite $\delta^{18}O$ and carbonate $\delta^{18}O$ offset of 8 ‰ in Fig. 5, Figs. S23–24, and Dataset S4.

1.9 Global Climate Model (GCM) for the Turkana Miocene

To evaluate global climate change through the Miocene, we performed paleoclimate simulations using the fully coupled water isotope-enabled Community Earth System Model (iCESM) by the National Center for Atmospheric Research (NCAR) *(33)*. The iCESM is comprised of the Community Atmosphere Model version 5.3, the Parallel Ocean Program version 2, the Community Land Model version 4.0, the River Transport Model, and the Los Alamos Sea Ice Model version 4. Water isotopic ratios ($\delta^{18}O$ and δD) are incorporated into each component as hydrologic cycle tracers that track water fluxes and isotopic fractionation (*33*). Simulations were conducted using a f19_g16 (1.9x2.5° atmosphere, 1° ocean) resolution and B1850C5CN component set. This relatively coarse resolution allowed efficient equilibration of our earth system simulations and was appropriate given the coarse nature of paleoclimate boundary conditions,

notably the paleogeography. Previous studies have demonstrated that CESM1.2 has a higher climate sensitivity than its predecessors (e.g., the Community Climatic System Model; CCSM) and does an excellent job of simulating past cold and warm climates *(34-36)*.

For this study, Miocene simulations were completed each with age-specific boundary conditions (e.g., atmospheric CO_2 levels, geography, bathymetry, surface elevations, surface types, glacial ice, aerosols, and solar luminosity) for the early and middle Miocene *(36)*. Two simulations were run with CO_2 levels of 280 and 400 ppm, respectively, to evaluate the climate sensitivity to atmospheric CO_2. A third simulation was run at 400 ppm under an orbital configuration (eccentricity = 0.054, obliquity = 24.5, precession = 270) producing high Northern Hemisphere summer insolation and large seasonal insolation variation. In this work we refer to the ordinary 400 ppm simulation as the “control” run (modern configuration: eccentricity = 0.0167, obliquity = 24.45, precession = 90 with perihelion during austral summer) and to high Northern Hemisphere seasonality as the “high insolation” run. The 280 and 400 ppm simulations were each run for more than 1500 years after which the top of the atmosphere energy balance is <0.2 Wm-2, indicating that the runs are near equilibrium, and have global average temperatures of 18.9 (280 ppm) and 19.9°C (400 ppm), respectively. The high insolation simulation was branched from the 400 pm run and integrated for 200 years. Results were averaged over the last 100 years of the 280 and 400 ppm simulations and over the last 50 years of the high insolation. In the main text, we focus on the results for the 400 ppm CO_2 simulations. Reducing CO_2 to 280 ppm causes a systematic decrease in monthly precipitation and $\delta^{18}O$ over Kalodirr but has little effect on the seasonality or seasonal range.

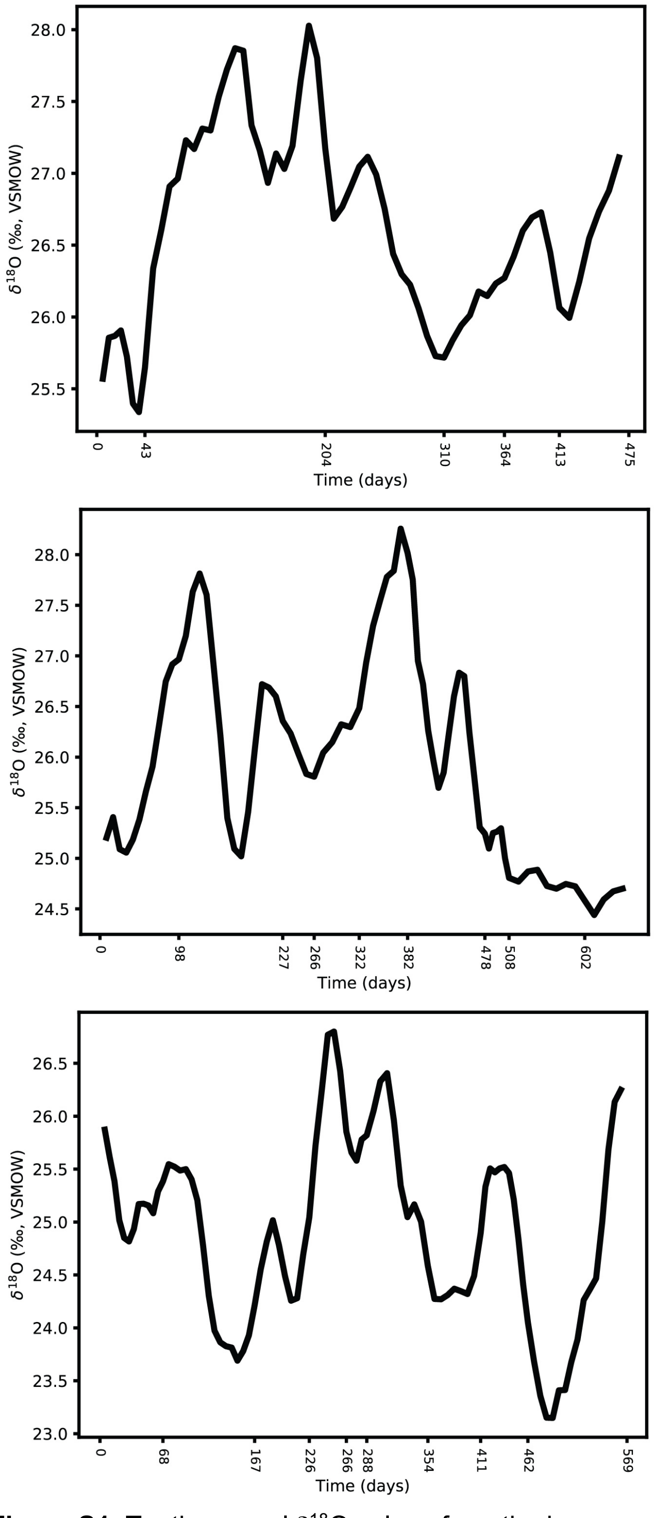

Figure S1. Tooth enamel $\delta^{18}O$ values from the innermost enamel along the enamel-dentine junction (EDJ) of the M1 (top), M2 (middle), and M3 (bottom) of baboon 73261. Days along the x-axis indicate the progressive formation of each crown.

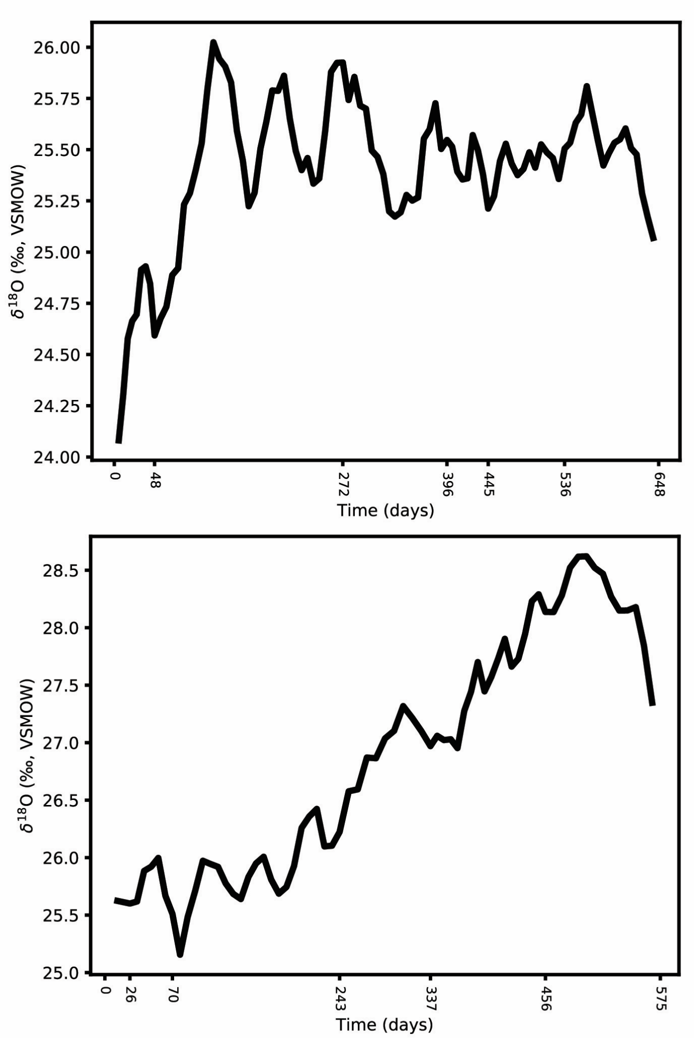

Figure S2. Tooth enamel $\delta^{18}O$ values from the M1 (top) and M2 (bottom) of baboon 73436. Days along the x-axis indicate the progressive formation of each crown. The M3 was not sampled as it was too immature to return reliable measurements.

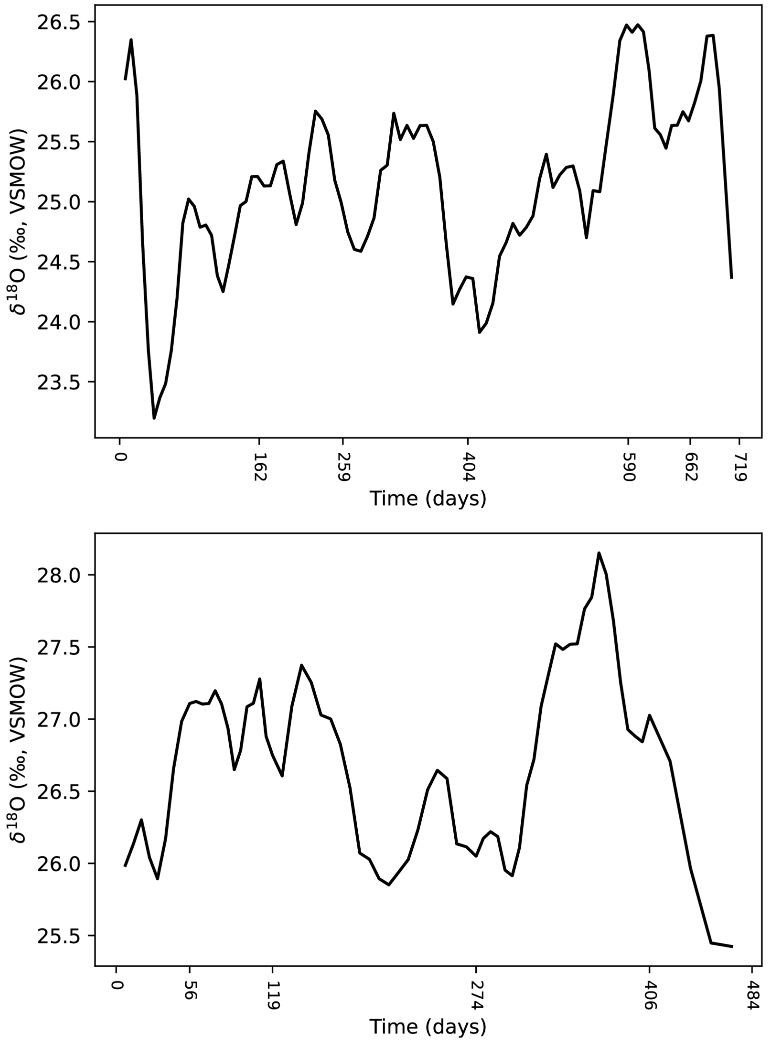

Figure S3. Tooth enamel $\delta^{18}O$ values from the M2 (top) and M3 (bottom) of baboon HT 17-02. Days along the x-axis indicate the progressive formation of each crown.

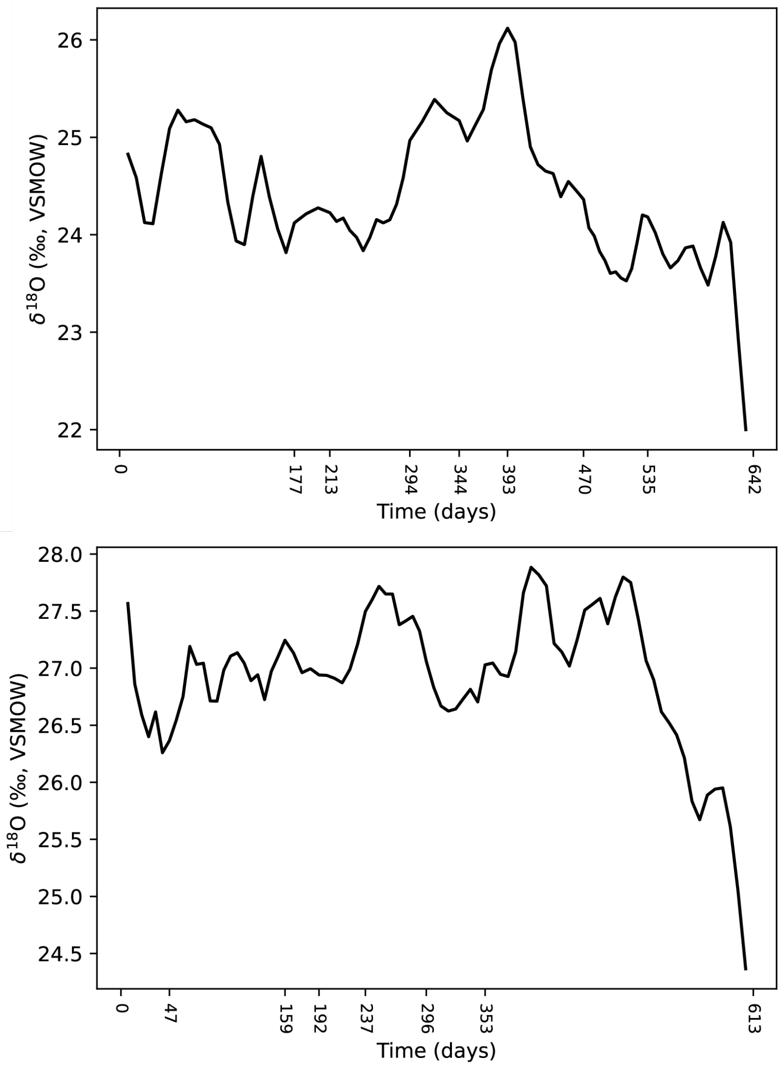

Figure S4. Tooth enamel $\delta^{18}O$ values from the M1 (top) and M2 (bottom) of baboon HT 18-02. Days along the x-axis indicate the progressive formation of each crown.

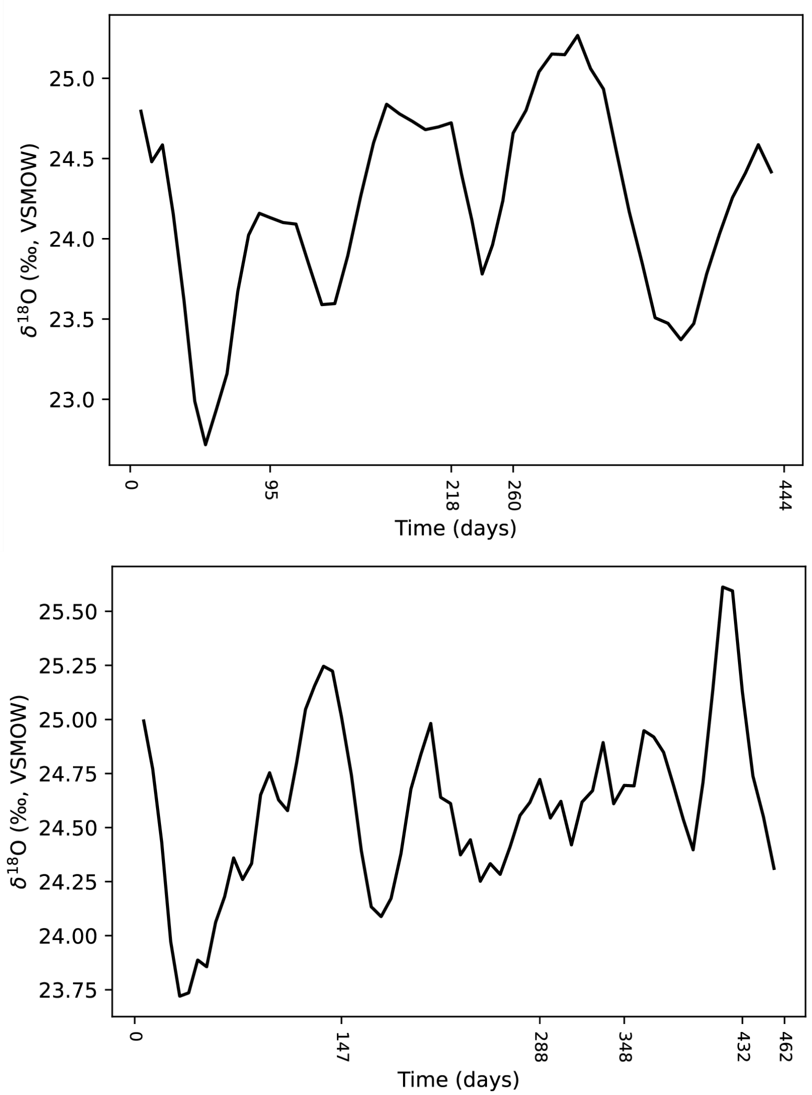

Figure S5. Tooth enamel $\delta^{18}O$ values from the M1 (top) and M2 (bottom) of baboon HT 19-02. Days along the x-axis indicate the progressive formation of each crown.

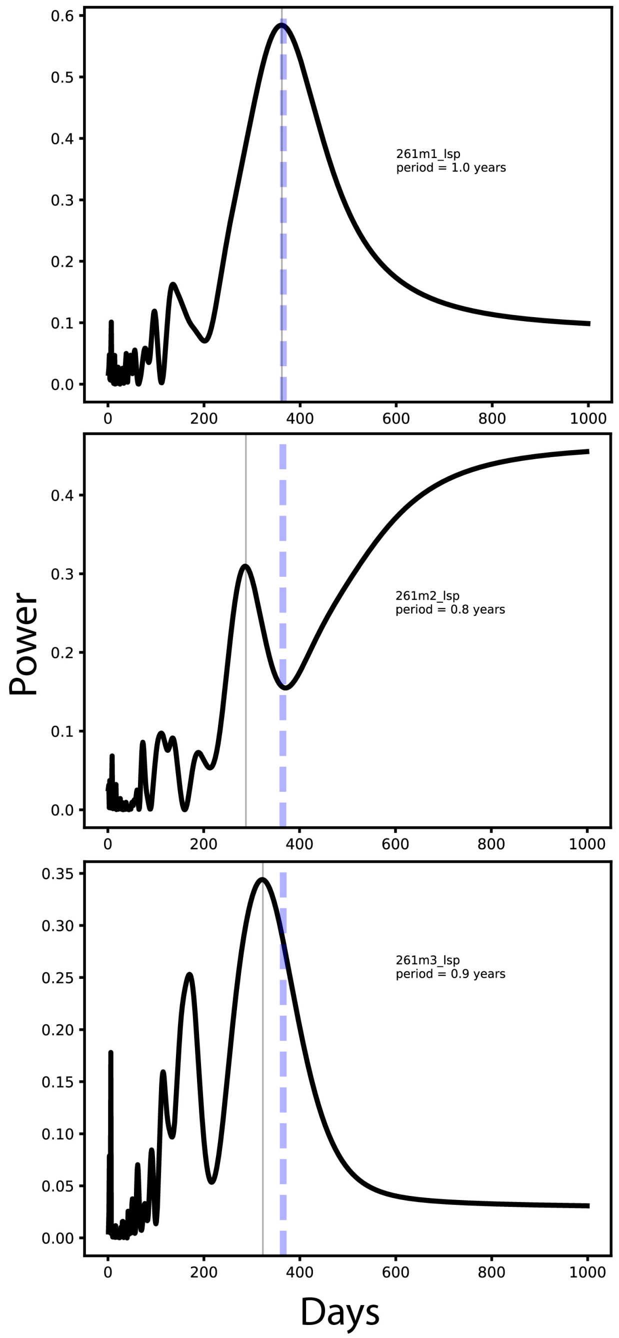

Figure S6. Inferred seasonality of $\delta^{18}O$ values from the M1 (top), M2 (middle), and M3 (bottom) of baboon 73261. Lomb-Scargle periodograms show potential periods in days (x-axis) against period power (y-axis), where higher values on the y-axis indicate underlying sine-wave periods that produce, contribute to, or explain $\delta^{18}O$ value oscillations. For example, Lomb-Scargle periodograms of rainfall data in a location with a single, clear and invariable monsoon at the same time every year would show a single peak at 1.0 years, while a periodogram analyzing rainfall with a monsoon every 6 months would show peaks at 0.5 and 1.0 years. See methods for further explanation. Best-fit periodicities are shown as light gray lines, whereas annual periodicities are indicated by blue dashed lines. Inferred frequencies are 1.0 years for the M1, 0.8 years for the M2, and 0.9 years for the M3, which also records an approximately 6-month peak, possibly reflecting the semiannual Ethiopian monsoon. Increasing powers at very high periods in some graphs are an artifact of limited sampling length within teeth.

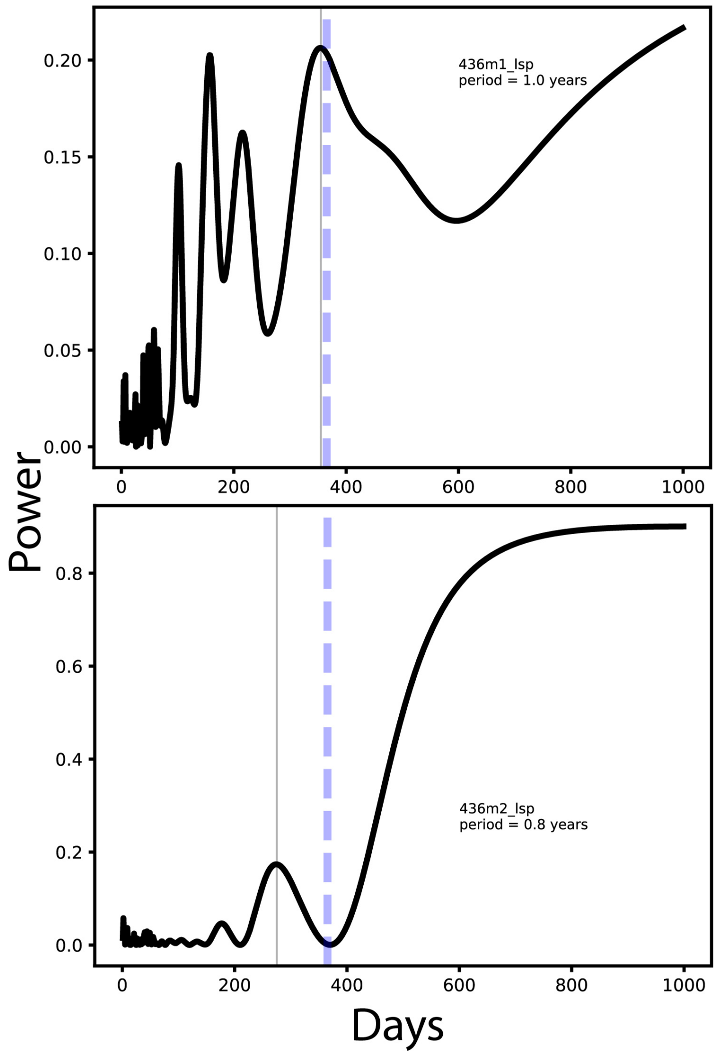

Figure S7. Lomb-Scargle periodograms show potential periods in days (x-axis) against period power (y-axis) for the M1 (top) and M2 (middle) of baboon 73436. Best-fit periodicities are shown as light gray lines, whereas annual periodicities are indicated by blue dashed lines. Though signals are not as strong as baboon 73261, this analysis finds an inferred frequency of 1.0 years for the M1, and 0.8 years for the M2.

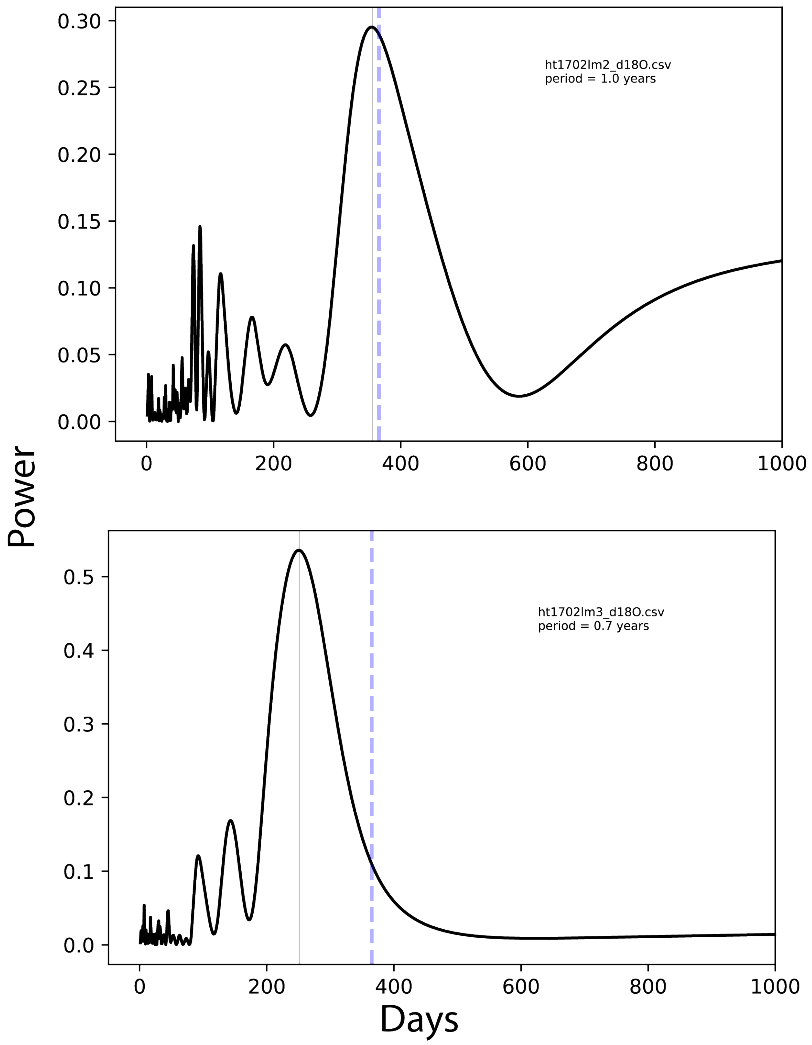

Figure S8. Lomb-Scargle periodograms show potential periods in days (x-axis) against period power (y-axis) for the M2 (top) and M3 (middle) of baboon HT 17-02. Best-fit periodicities are shown as light gray lines, whereas annual periodicities are indicated by blue dashed lines. The periodogram for the M2 indicates a robust annual periodicity, whereas the periodicity for the M3 is subannual.

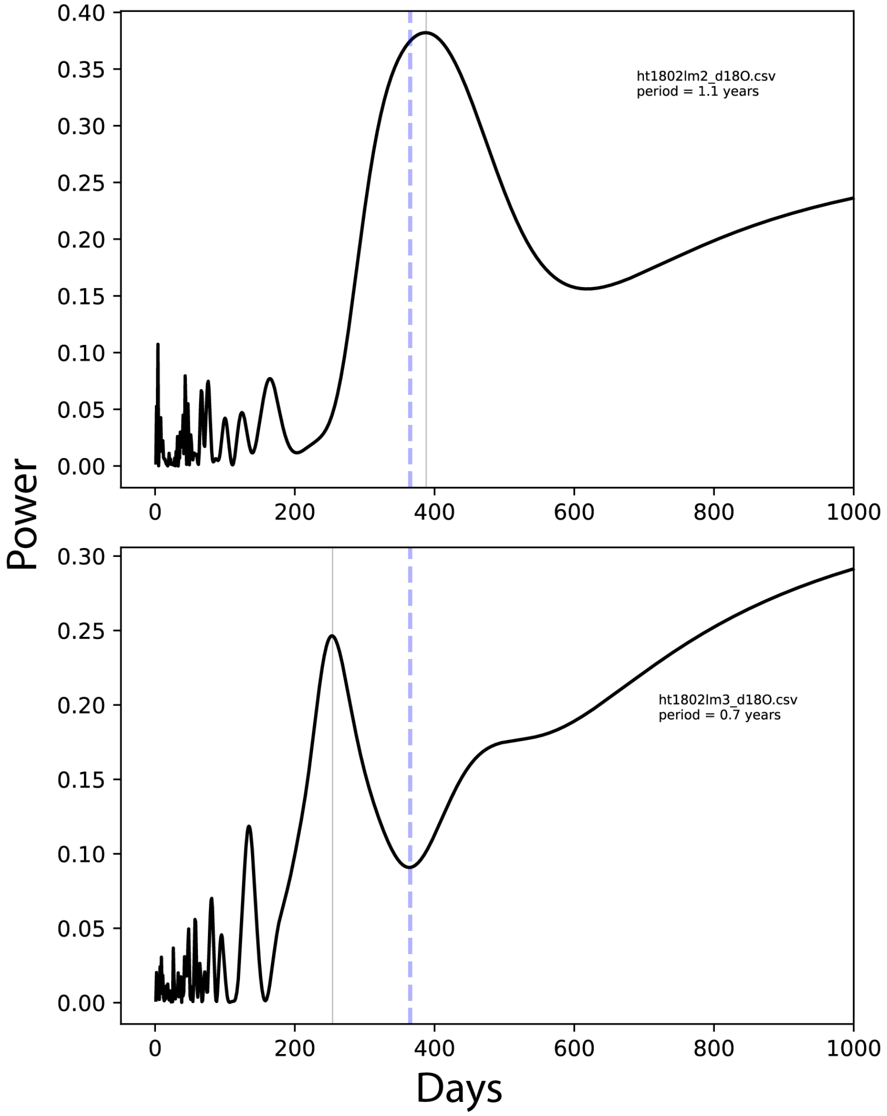

Figure S9. Lomb-Scargle periodograms show potential periods in days (x-axis) against period power (y-axis) for the M2 (top) and M3 (middle) of baboon HT 18-02. Best-fit periodicities are shown as light gray lines, whereas annual periodicities are indicated by blue dashed lines. The periodogram for the M2 indicates a robust annual periodicity, whereas the periodicity for the M3 is subannual.

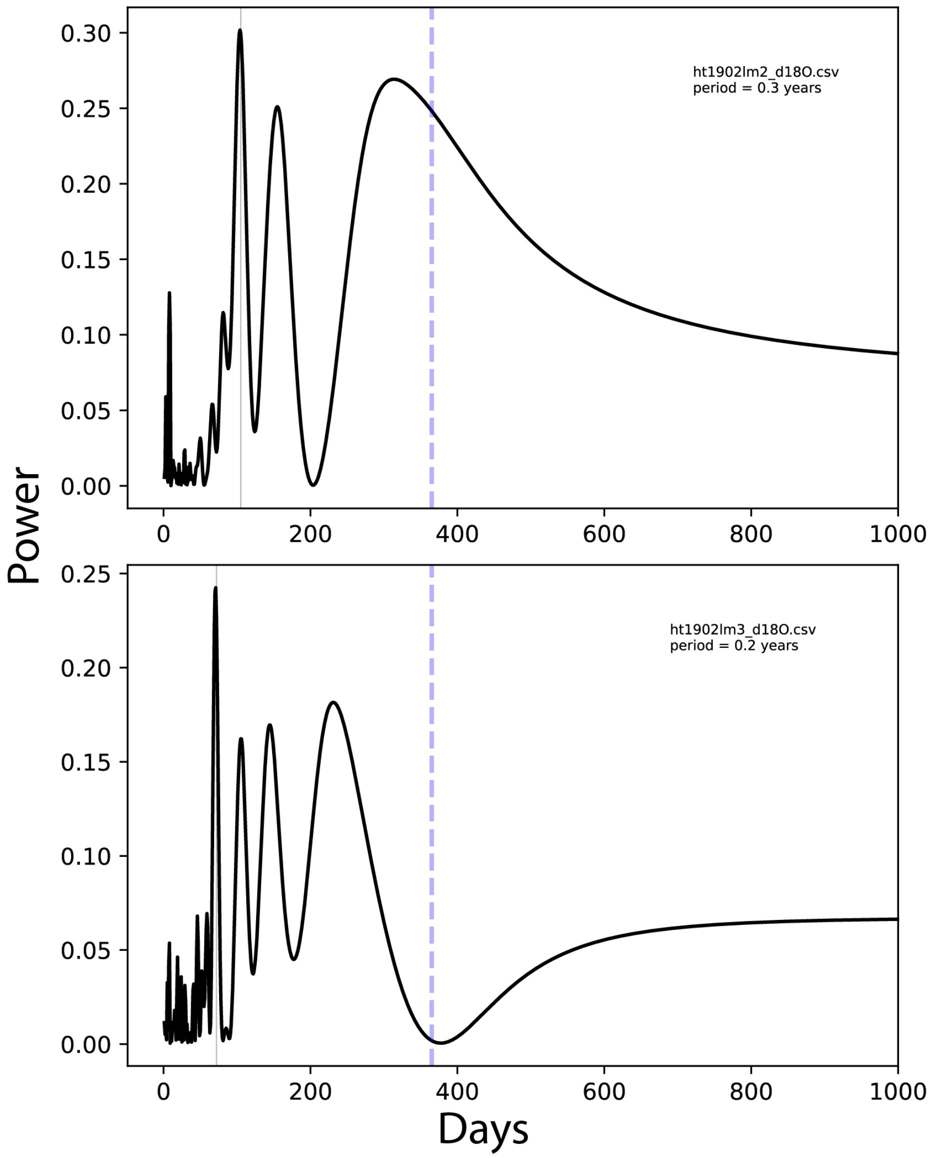

Figure S10. Lomb-Scargle periodograms show potential periods in days (x-axis) against period power (y-axis) for the M2 (top) and M3 (middle) of baboon HT 19-02. Best-fit periodicities are shown as light gray lines, whereas annual periodicities are indicated by blue dashed lines. Neither the M2 nor the M3 show clear 6- or 12-month periodicity.

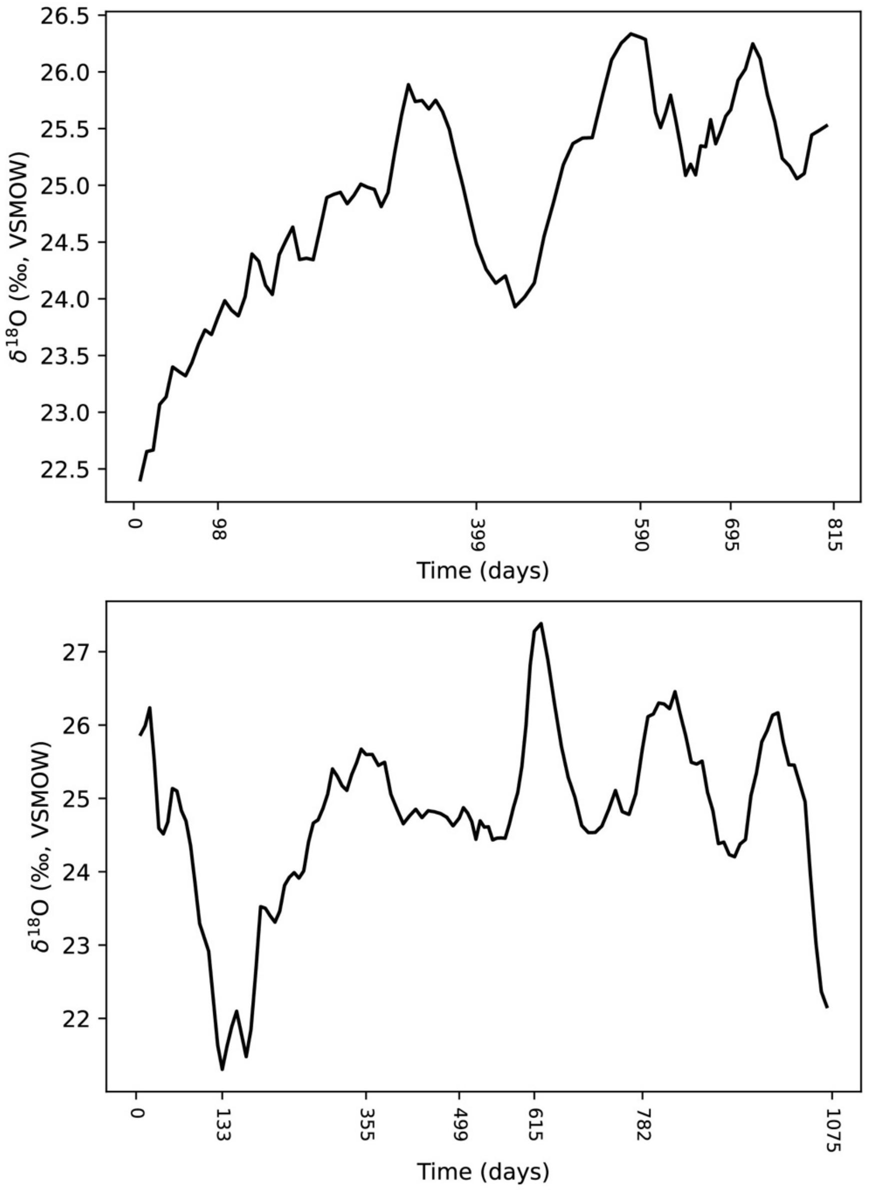

Figure S11: Tooth enamel $\delta^{18}O$ values from the LM2 of gelada HKU 0237 (top), and the M3 of gelada HKU 0243 (bottom), from the Debre highlands in Ethiopia. Days along the x-axis indicate the progressive formation of each crown.

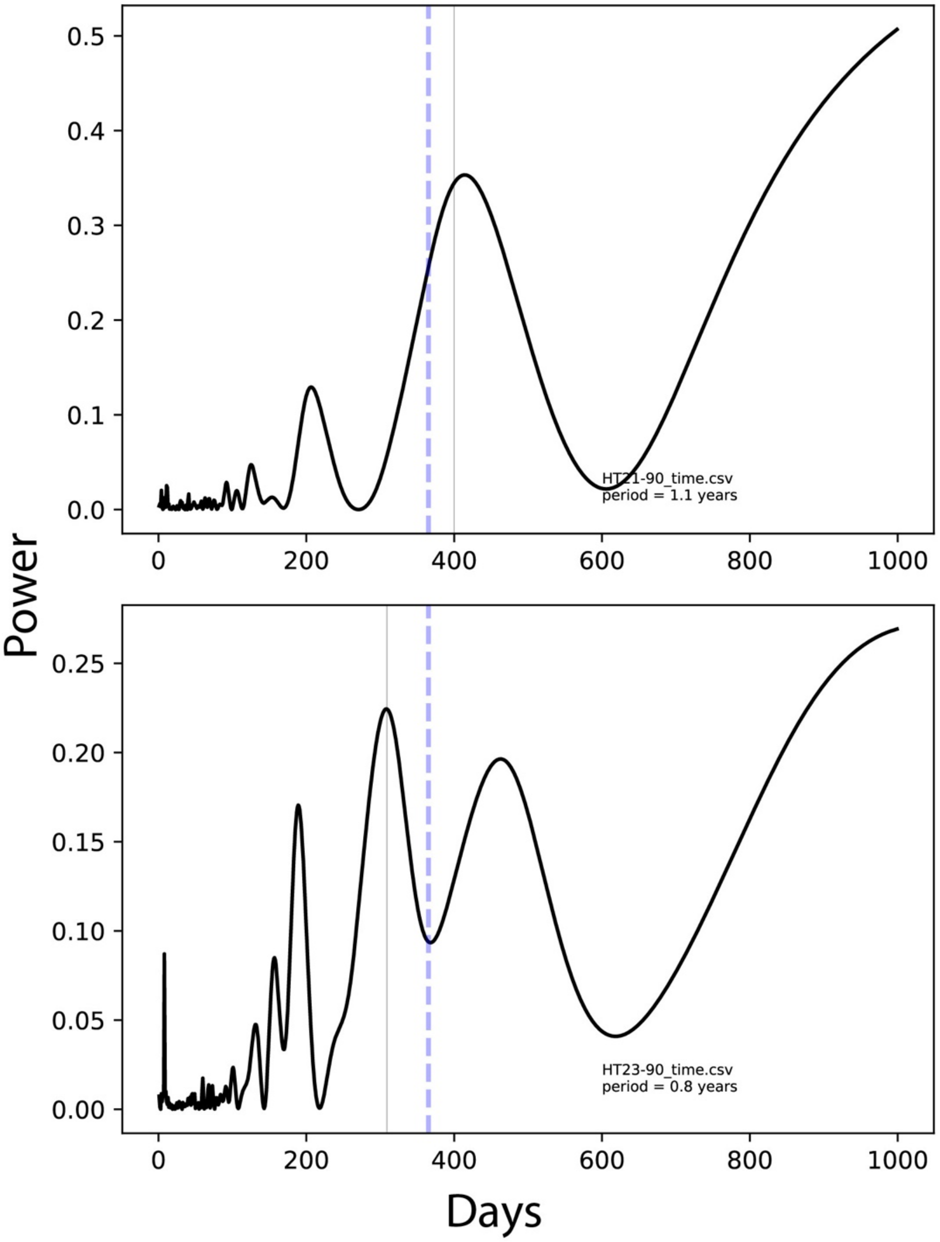

Figure S12: Inferred seasonality of $\delta^{18}O$ values from Debre highland geladas. Lomb-Scargle periodograms show potential period in days (x-axis) against periods power (y-axis) *for Theropithecus gelada* HKU 0237 (top) and HKU 0243 (bottom). Inferred frequencies are annual, with 1.1-year periodicities for both specimens, but as with some Awash baboon teeth, strong c. 6-month peaks are also seen for both specimens here. Best-fit periodicities are shown as light gray lines, whereas annual periodicities are indicated by blue dashed lines.

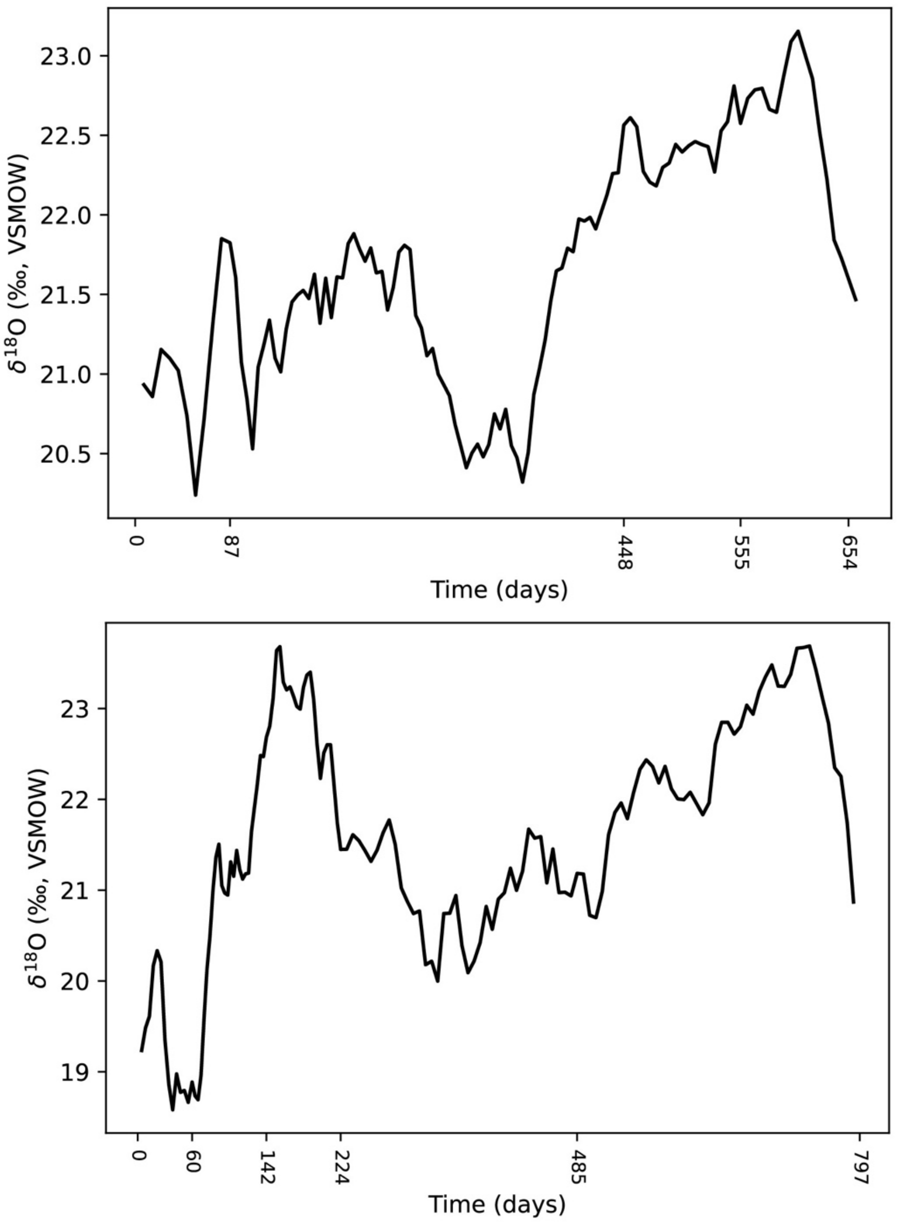

Figure S13: Tooth enamel $\delta^{18}O$ values from the LM1 (top) and LM2 (bottom) of Bushenyi *Papio anubis* baboon U9. Days along the x-axis indicate the progressive formation of each crown.

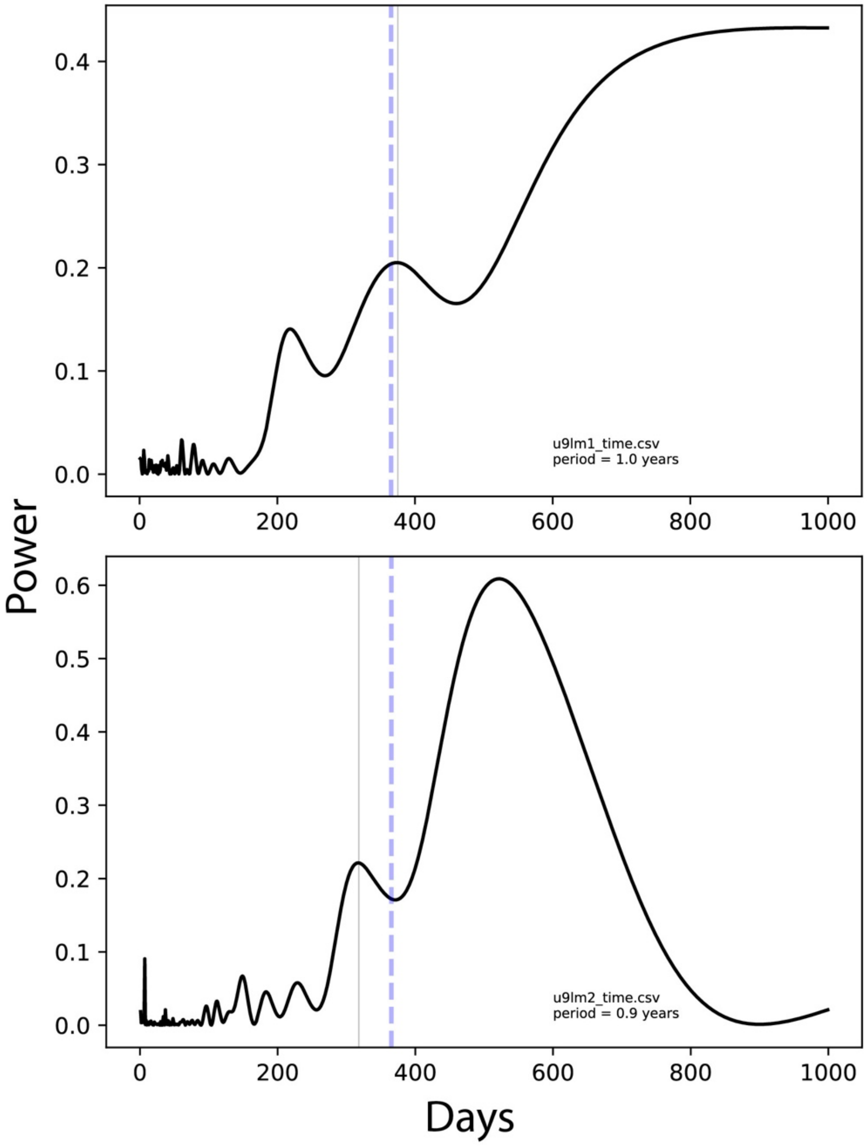

Figure S14: Inferred seasonality of $\delta^{18}O$ values from of Bushenyi *Papio anubis* baboon U9. Lomb-Scargle periodograms show potential period in days (x-axis) against periods power (y-axis) for *Papio anubus* specimens U9 LM1 (top) and LM2 (bottom). Peaks are seen at 0.9 and 1.0 years for both specimens, but are annual peak power is weak compared to other frequencies. Best-fit periodicities are shown as light gray lines, whereas annual periodicities are indicated by blue dashed lines.

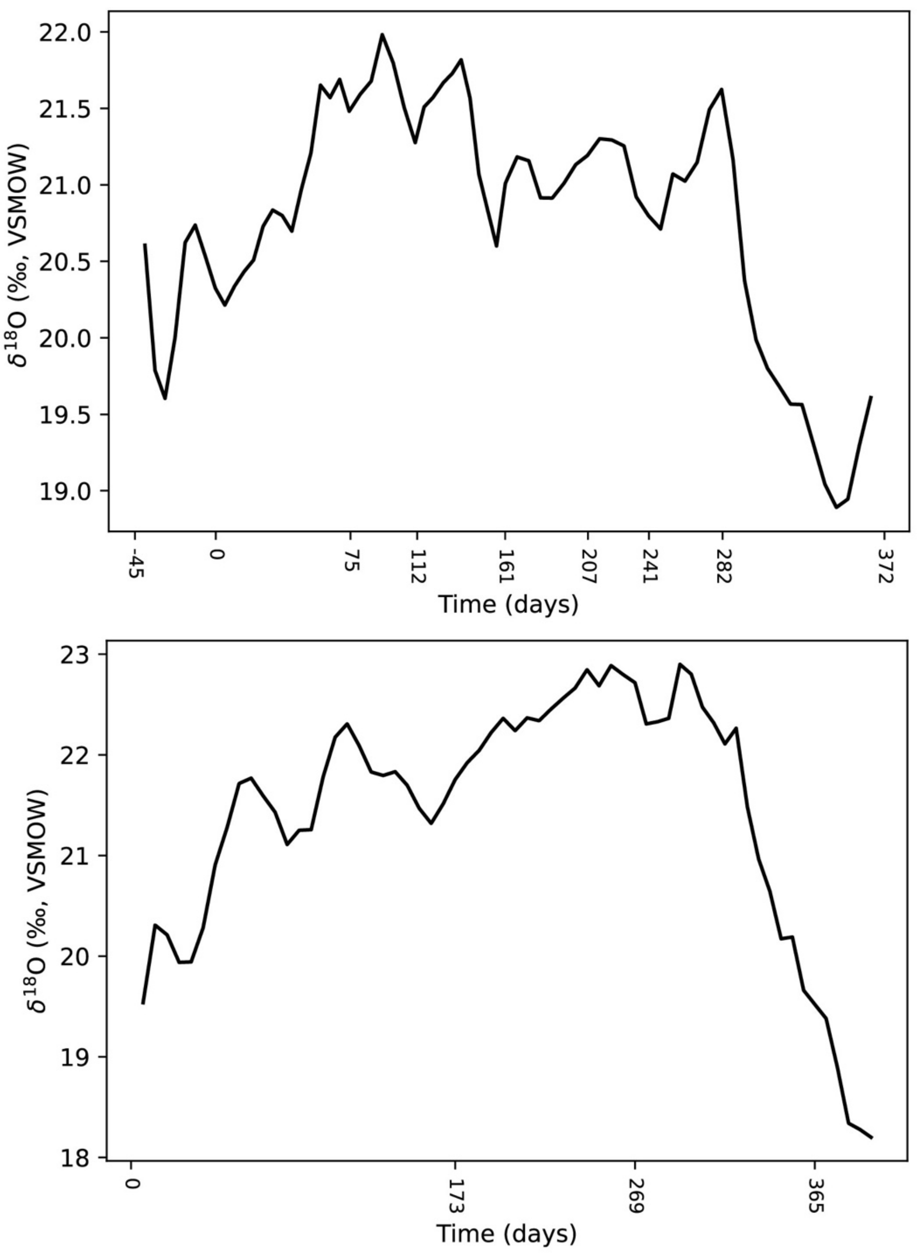

Figure S15: Tooth enamel $\delta^{18}O$ values of Bushenyi *Papio anubis* individual U10 LM1 (top) and lower canine (bottom). Days along the x-axis indicate age for the M1 above, and the progressive formation of the enamel crown for the canine below.

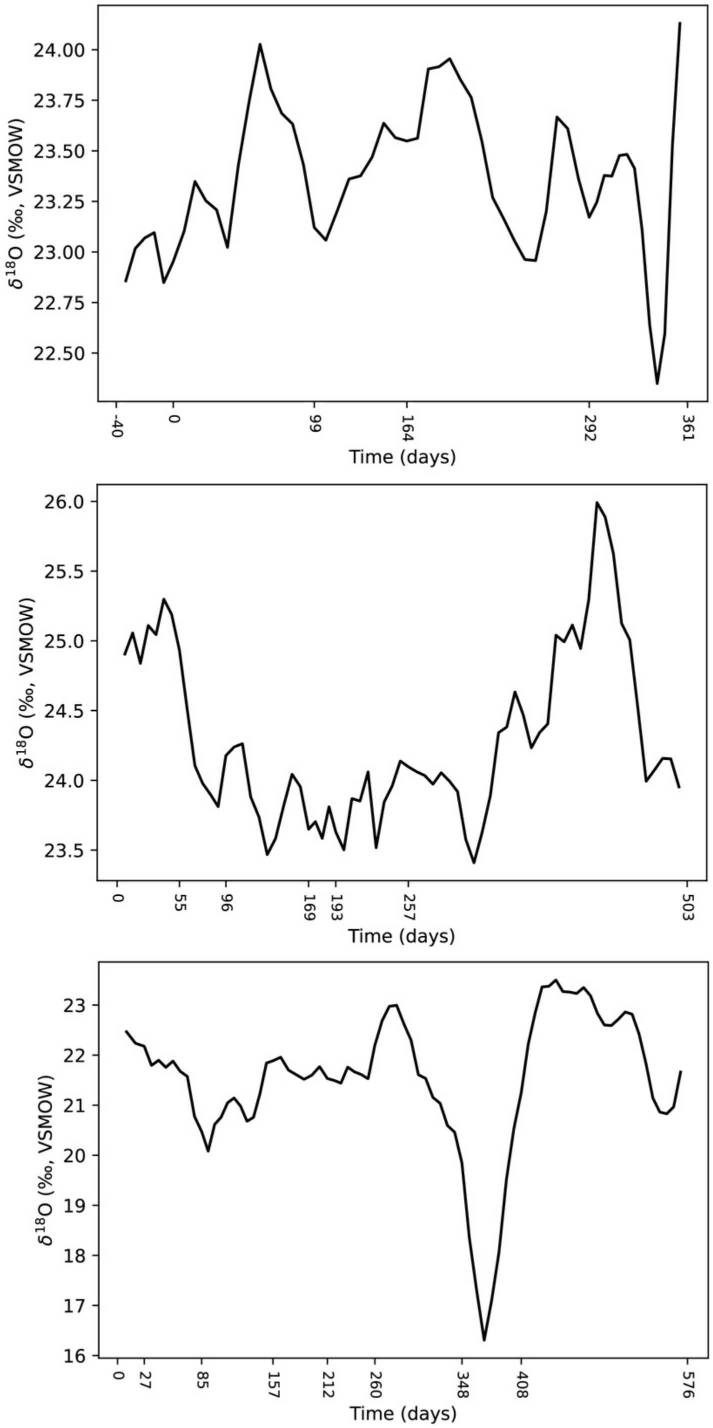

Figure S16: Tooth enamel δ^{18}O values from the LM1 (top), LM2 (middle) and LM3 (bottom) of Bushenyi Distrcit *Chlorocebus tantalus* HT 06-02. Formation times are relatively short, but the longest forming tooth (M3) includes a brief but steep decline in δ^{18}O values that distinguishes this tooth from the other tantalus monkey sampled here; see main text and Fig. S13 for more information. Time on the x-axis indicates animal age for the M1 above, and the timing of tooth formation for the M2 and M3 below.

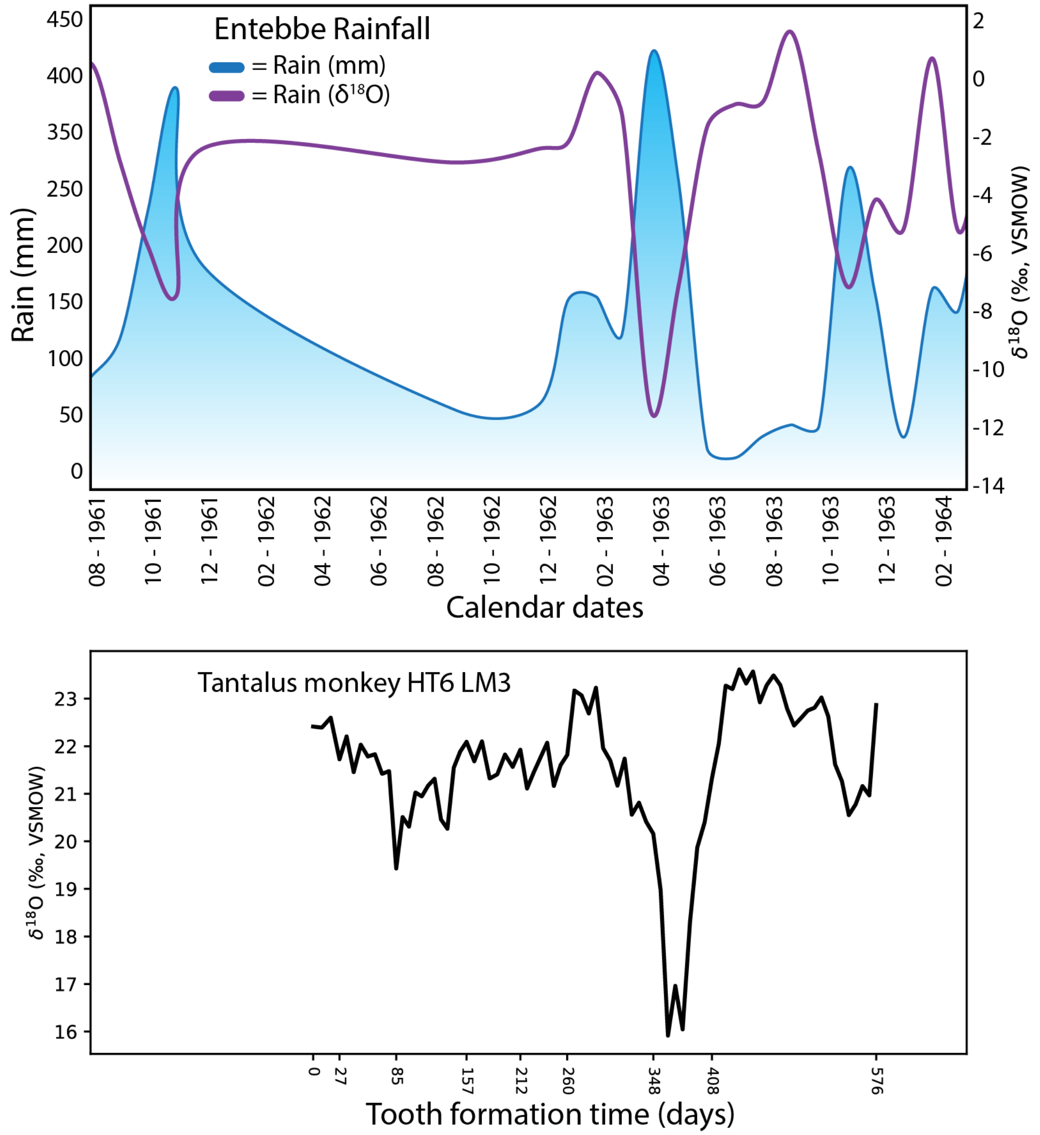

Figure S17. Rainfall records in Entebbe in 1963 *(20)*, alongside the Bushenyi District tantalus monkey HT 06-02 M3 $\delta^{18}O$ profile that formed during approximately the same period (the animal was collected in 1965). At Entebbe, periods of high rain correspond to low rainfall $\delta^{18}O$ values, as expected. A long drought, lasting the entirety of 1962, was broken by rains in April 1963 that were reported in international newspapers, and accompanied by the lowest rainfall $\delta^{18}O$ values measured over the next 60 years. This event is consistent with the very low $\delta^{18}O$ values measured from the M3.

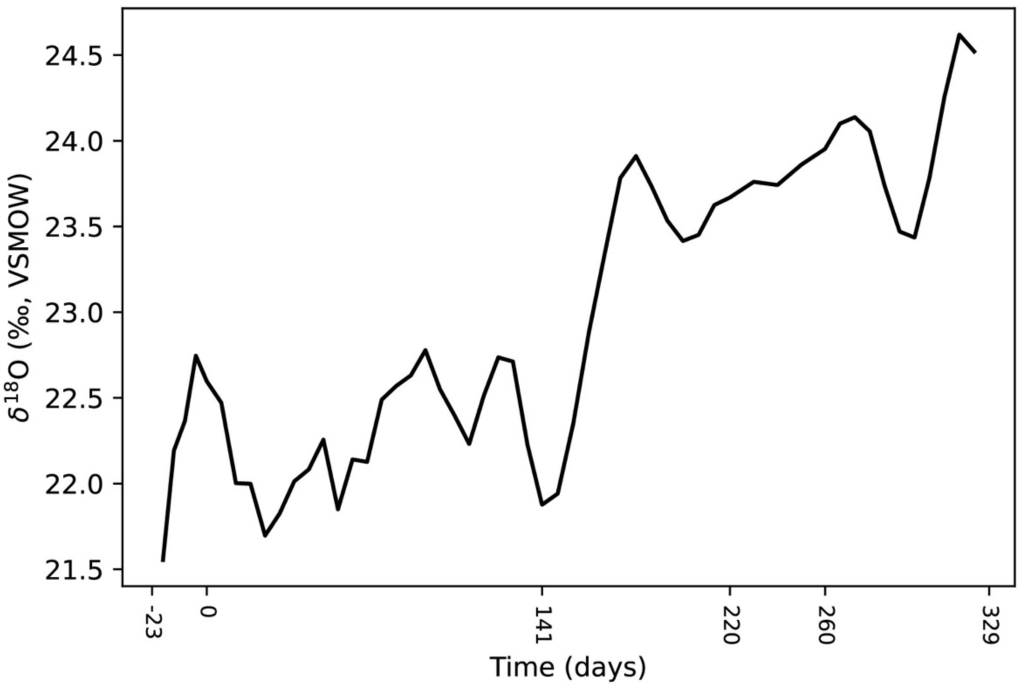

Figure S18 Tooth enamel $\delta^{18}O$ values from the M1 of *Chlorocebus tantalus* HT 07-02 from Bushenyi District in Uganda. Time in days represents the age of this animal.

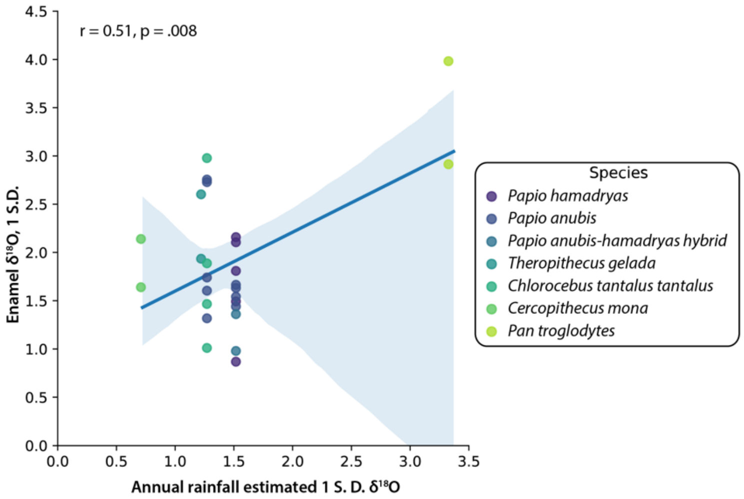

Figure S19. Relationship between rainfall and primate $\delta^{18}O$ variability for teeth examined in this study. The relationship between estimated 1 S.D. of estimated monthly rainfall $\delta^{18}O$ values (‰) on the x-axis *(20-23)* is compared to 1 S.D. of intra-tooth profiles on the y-axis. The relationship is positive but inconsistent.

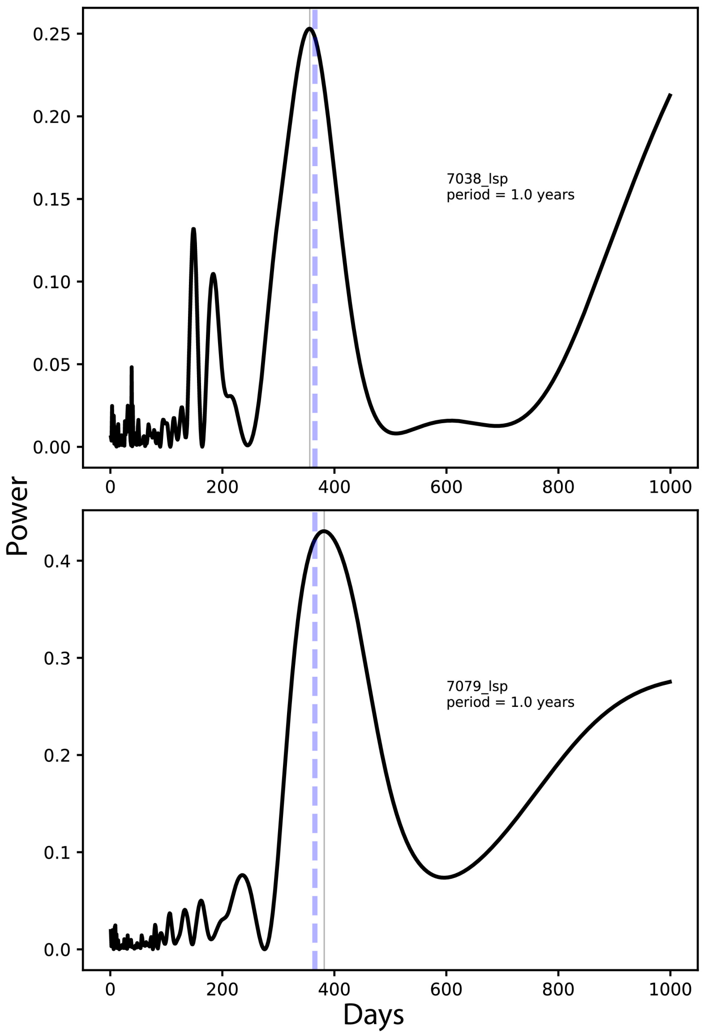

Figure S20. Inferred seasonality of $\delta^{18}O$ values from Liberian chimpanzees. Lomb-Scargle periodograms show potential period in days (x-axis) against periods power (y-axis) for *Pan troglodytes verus* specimens 7038 (top) and 7079 (bottom). Inferred frequencies are annual, with 1.0-year periodicities for both specimens. Best-fit periodicities are shown as light gray lines, whereas annual periodicities are indicated by blue dashed lines.

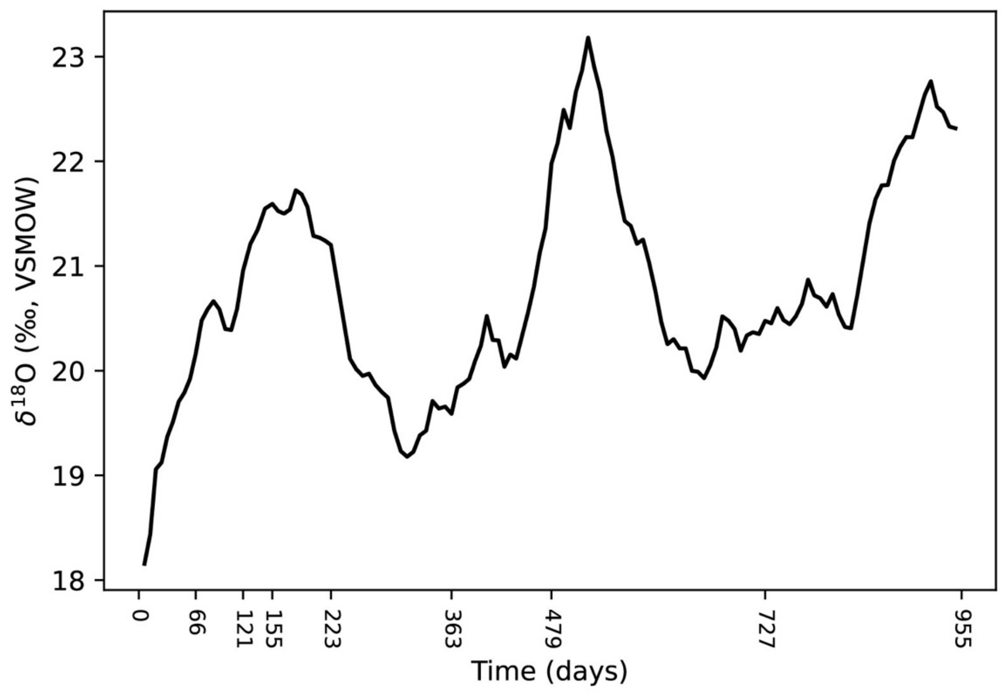

Figure S21: Tooth enamel δ^{18}O values from the lower canine of *Cercopithecus mona* individual HT 01-10 from Benin.

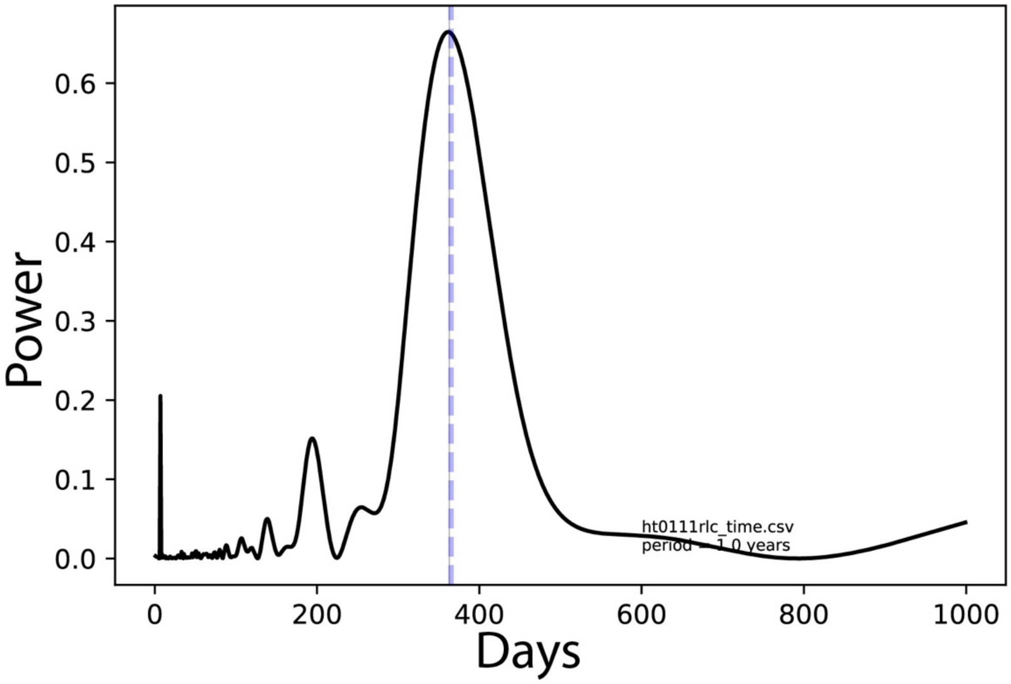

Figure S22: Inferred seasonality of $\delta^{18}O$ values from the lower canine of *Cercopithecus mona* individual HT 01-10. Lomb-Scargle periodograms show potential period in days (x-axis) against periods power (y-axis); in this cass the inferred frequency is clearly annual, with 1.0-year periodicity explaining most observed signal. Best-fit periodicities are shown as light gray lines, whereas annual periodicities are indicated by blue dashed lines.

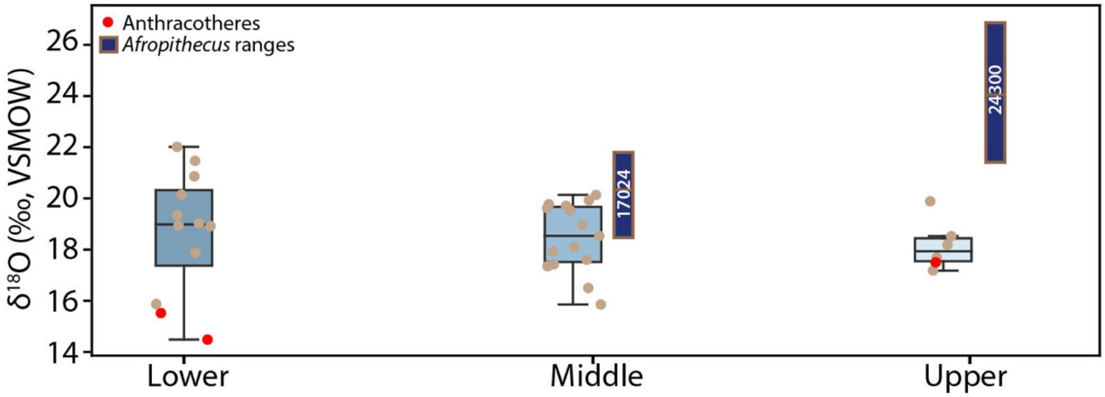

Figure S23. Boxplots for $\delta^{18}O$ data from Kalodirr herbivores reveal greater variability for older specimens (lower in stratigraphic section, including from the Kanukurinya site), and less variability for younger specimens (upper portion of section), possibly the result of sample paucity in the upper part of the section. $\delta^{18}O$ from each individual sampled are shown with light brown circles, anthracotheres are indicated with red circles. Oxygen isotope ranges for each *Afropithecus* individual are shown in dark blue adjacent to the portion of the section from which they were sampled. Herbivore teeth from Kalodirr indicate that coarse population-scale $\delta^{18}O$ variability decreases upwards throughout the depositional sequence, with higher variance in the low stratigraphic section compared to the middle (F-test of variance, $p = 0.045$) and high ($p = 0.060$) sections. This result is not explained by taxonomic diversity since six families are recorded in each sequence.

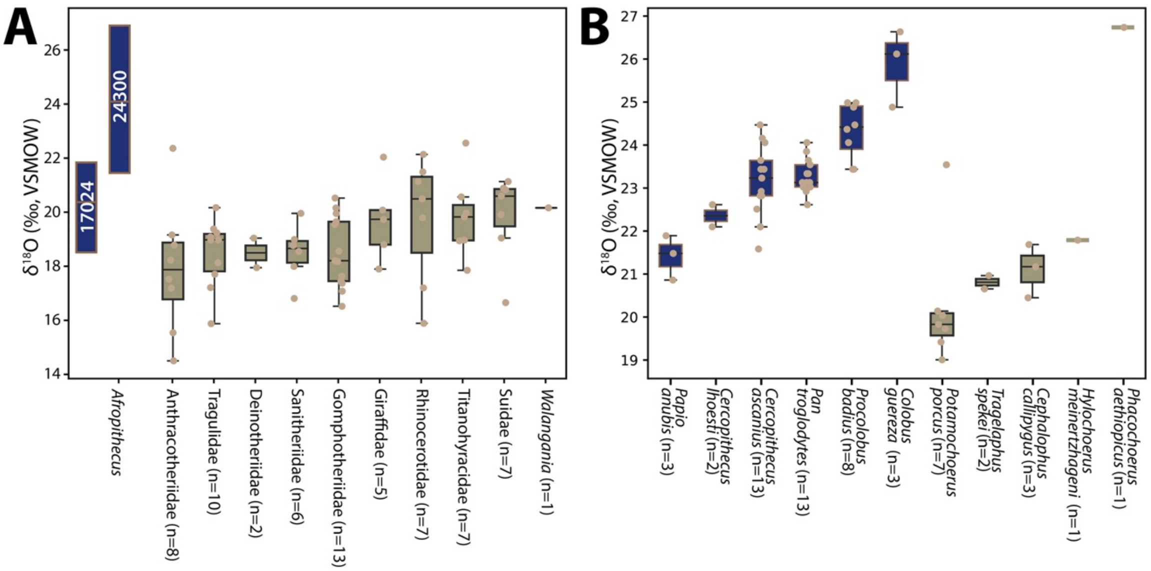

Figure S24. Primate $\delta^{18}O$ values are generally elevated compared to terrestrial herbivores in Miocene and modern African ecosystems. **(A)** $\delta^{18}O$ from two *Afropithecus* specimens (dark blue) are enriched compared to terrestrial herbivores (light brown) from Kalodirr. **(B)** Similar isotopic separation and values are observed in Kibale National Park, Uganda *(37)*, where primate $\delta^{18}O$ values (dark blue) are higher than terrestrial herbivores (light brown), with the exception of the terrestrial baboon (*Papio anubis*).

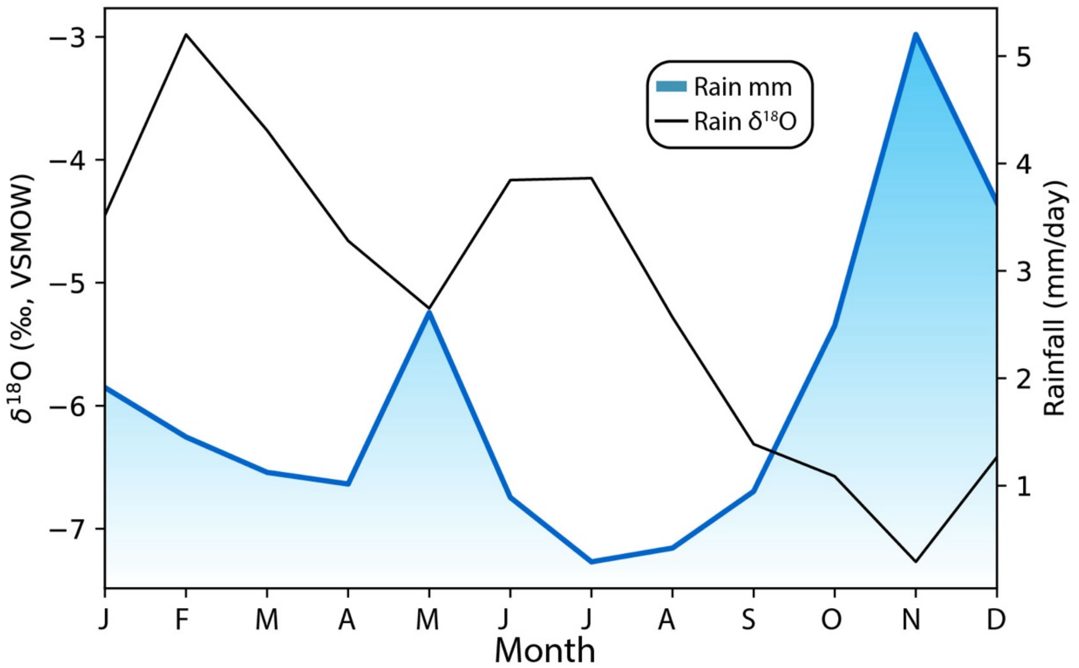

Figure S25. Earth system model daily rainfall (blue) and rain $\delta^{18}O$ values (black) simulated using pre-industrial conditions in the Turkana region around Kalodirr. As in modern systems, rainfall amount and stable isotope values are anticorrelated.

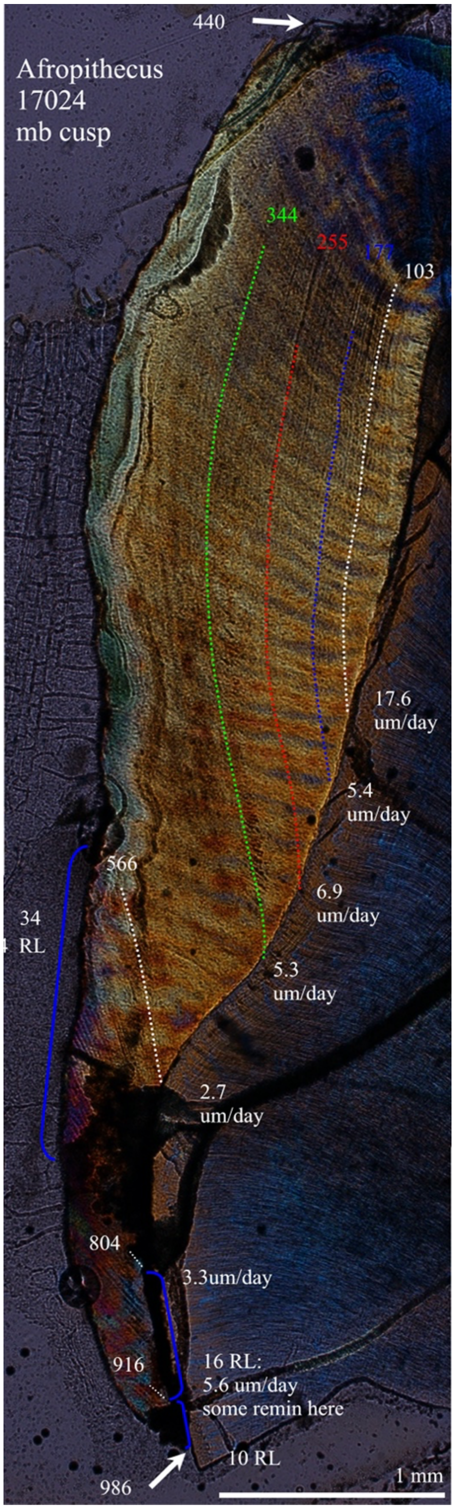

Figure S26. Developmental map of *Afropithecus* specimen KNM-WK 17024 showing the progressive development of the tooth in days and the speed of enamel extension in microns/day. Diagenetic modification between 566 and 804 days prohibited continuous fine-scaled sampling along the EDJ. RL = Retzius lines, long-period growth increments that can be counted in subsurface enamel and traced back to the EDJ to determine extension rates in lateral and cervical enamel.

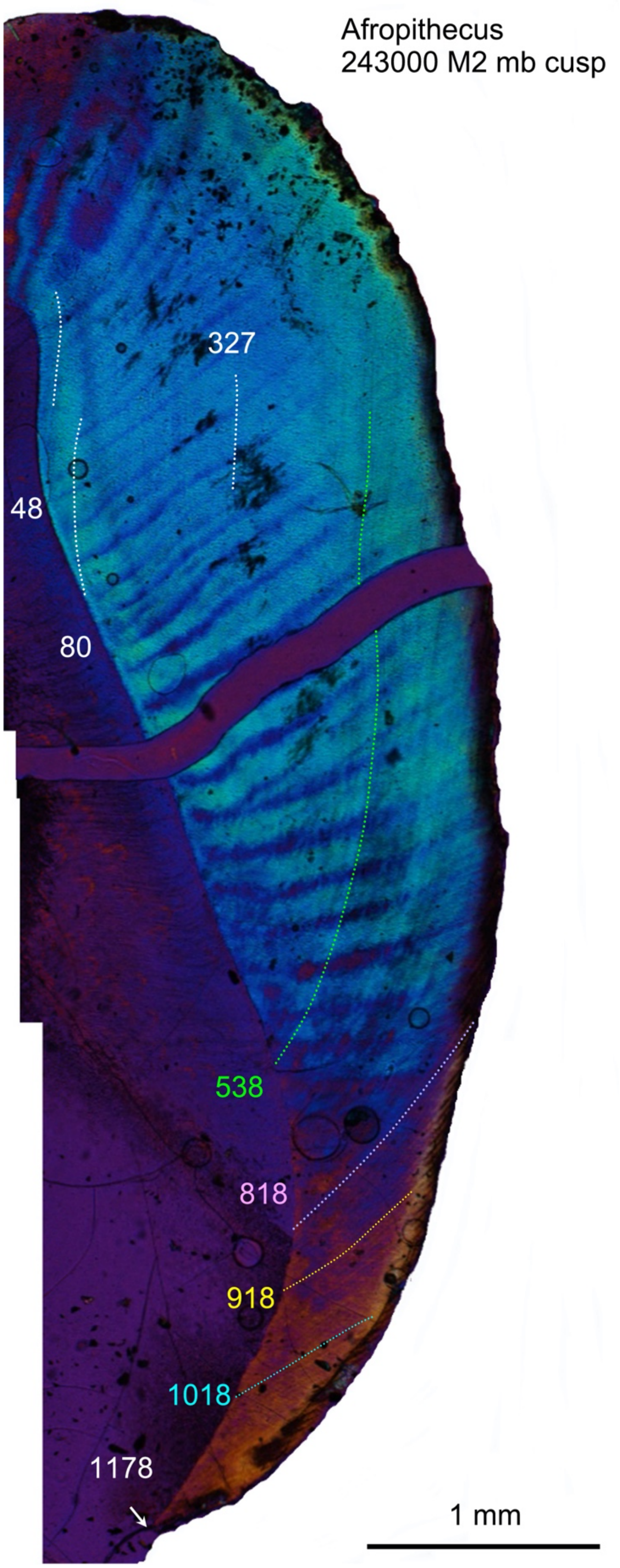

Figure S27. Developmental map of *Afropithecus* specimen KNM-WK 24300 showing the progressive development of the tooth in days.

Datasets S1-S4 (separate file). Legends for these datasets are included in the dataset file.

SI References

1. G. L. Moritz, et al., Baboons, water, and the ecology of oxygen stable isotopes in an arid hybrid zone. *Physiol. Biochem.* **85**(5), 421-30 (2012).
2. T. M. Smith, M. Arora, M. Bharatiya, W. Dirks, C. Austin, Brief communication: Elemental models of primate nursing and weaning revisited. *Am. J. Biol. Anthropol.* (*In Press*).
3. J. E. Phillips-Conroy, C. J. Jolly. Changes in the structure of the baboon hybrid zone in the Awash National Park, Ethiopia. *Am. J. Phys. Anthropol.* **71**(3), 337-50 (1986).
4. W. Dirks, D. J. Reid, C. J. Jolly, J. E. Phillips-Conroy, F. L. Brett, Out of the mouths of baboons: stress, life history, and dental development in the Awash National Park hybrid zone, Ethiopia. *Am. J. Phys. Anthropol.* **118**(3), 239-52 (2002).
5. U. Nagel, A comparison of anubis baboons, hamadryas baboons and their hybrids at a species border in Ethiopia. *Folia Primatol.* **19**(2-3), 104-65 (1973).
6. C. H. Remien, F. R. Adler, L. A. Chesson, L. O. Valenzuela, J. R. Ehleringer, T. E. Cerling. Deconvolution of isotope signals from bundles of multiple hairs. Oecologia. 175(3):781-9 (2014).
7. D. R. Swindler, A. D. Beynon. *Theropithecus*: The development and microstructure of the dentition of Theropithecus. In *Theropithecus*: the rise and fall of a genus, Edited by Nina Jablonski (University of Western Australia, Perth, 1993).
8. B. G. Shefine. Analysis of Meteorological Drought Using SPI and Large-Scale Climate Variability (ENSO)-A Case Study in North Shewa Zone, Amhara Regional State, Ethiopia. *Hydrol Current Res*. **9**(307):2 (2018).
9. T. Iwamoto, R. I. Dunbar. Thermoregulation, habitat quality and the behavioural ecology of gelada baboons. *J. Anim. Ecol.* 357-66 (1983)
10. W. Dirks, D. J. Reid, C. J. Jolly, J. E. Phillips-Conroy, F. L. Brett. Out of the mouths of baboons: Stress, life history, and dental development in the Awash National Park hybrid zone, Ethiopia. *Am. J. Phys. Anthropol.*, **118**(3), 239–252 (2002). https://doi.org/10.1002/ajpa.10089
11. T. E Rowell,. Forest living baboons in Uganda. *J. Zool. Lond.*, **149**, 344–364 (1966).
12. T. R.Turner, C. A.Schmitt, J. D.Cramer, Savanna Monkeys: The Genus Chlorocebus (Cambridge University Press, 2019).
13. C. M. Hill. Conflict of interest between people and baboons: crop raiding in Uganda. *Int. J. Primatol.*, **21**(2), 299–315 (2000).
14. F. L Agmen, H. M. Chapman, M. Bawuro. Seed dispersal by tantalus monkeys (Chlorocebus tantalus tantalus) in a Nigerian montane forest. *Afr. J. Ecol.*, **48**(4), 1123–1128 (2009). https://doi.org/10.1111/j.1365-2028.1997.095-89095.x
15. R. McFarland, et al. Keeping cool in the heat: Behavioral thermoregulation and body temperature patterns in wild vervet monkeys. *Am. J. Phys. Anthropol.*, **171**(3), 407–418 (2020). https://doi.org/10.1002/ajpa.23962
16. Young, C., Bonnell, T. R., Brown, L. R., Dostie, M. J., Ganswindt, A., Kienzle, S., … Barrett, L. (2019). Climate induced stress and mortality in vervet monkeys. *R. Soc. Op. Sci.*, 6(11). https://doi.org/10.1098/rsos.191078
17. R. M. Goodwin. Behavior and ecology of the mona monkey in the seasonally dry Lama Forest, Republic of Bénin (CUNY Academic Works, 2017).
18. C. C. Smith, M. E. Morgan, D. Pilbeam, Isotopic ecology and dietary profiles of Liberian chimpanzees. *J. Hum. Evol.* **58**(1), 43-55 (2010).
19. G. W. Harley, Roads and trails in Liberia. *Geogr.* **29**(3), 447-60 (1939).
20. IAEA/WMO, "Global Network of Isotopes in Precipitation", The GNIP Database, http://www.iaea.org/water (2020).
21. G. J. Bowen, The Online Isotopes in Precipitation Calculator, version 3.1. http://www.waterisotopes.org (2022).
22. G. J. Bowen, J. Revenaugh, Interpolating the isotopic composition of modern meteoric precipitation. *Water Resources Res.* 39(10), 1299, (2003)

23. G. J. Bowen, L. I. Wassenaar, K. A. Hobson K. A. Global application of stable hydrogen and oxygen isotopes to wildlife forensics. *Oecologia* **143**, 337-348, doi:10.1007/s00442-004-1813-y (2003).
24. G. J. Bowen, Isoscapes: spatial pattern in isotopic biogeochemistry. *Ann. Rev. Earth Planet. Sci.* **38**, 161-87 (2010).
25. Y. Nan, F. Tian, H. Hu, L. Wang, S, Zhao, Stable isotope composition of river waters across the world. *Water* **11**(9):1760 (2019).
26. S. Terzer, L. I. Wassenaar, L. J. Araguás-Araguás, P. K. Aggarwal, Global isoscapes for δ18O and δ2H in precipitation: improved prediction using regionalized climatic regression models. *Hydrol. Earth Sys. Sci.* **17**(11), 4713-28 (2013).
27. S. A. Blumenthal, T. E. Cerling, K. L. Chritz, T. G. Bromage, R. Kozdon, J. W. Valley, Stable isotope time-series in mammalian teeth: In situ δ18O from the innermost enamel layer. *Geochim. Cosmochim. Ac.* **124**, 223-36 (2014).
28. T. M. Smith, et al. Williams, Wintertime stress, nursing, and lead exposure in Neanderthal children. *Sci. Adv.* **4**(10), eaau9483 (2018).
29. H. B. Boschetto, F. H. Brown, I. McDougall, Stratigraphy of the Lothidok Range, northern Kenya, and K/Ar ages of its Miocene primates. *J. H. Evol.* **22**, 47–71 (1992)
30. J. Oerlemans, Correcting the Cenozoic δ18O deep-sea temperature record for Antarctic ice volume. *Palaeogeogr. Palaeoclimatol. Palaeoecol.* **208**(3-4), 195-205 (2004).
31. B. S. Cramer, K. G. Miller, P. J. Barrett, J. D. Wright, Late Cretaceous–Neogene trends in deep ocean temperature and continental ice volume: Reconciling records of benthic foraminiferal geochemistry (δ18O and Mg/Ca) with sea level history. *J. Geophys. Res. Oceans* **116**, C12023 (2011).
32. M. J. Kohn, T. E. Cerling, Stable isotope compositions of biological apatite. *Rev. Mineral. Geochem.* **48**(1), 455-88 (2002).
33. E. Brady, et al. The Connected Isotopic Water Cycle in the Community Earth System Model Version 1. *J. Adv. Model. Earth Sys.* **11**(8), 2547–2566. (2019). https://doi.org/10.1029/2019MS001663
34. J. Zhu, C. J. Poulsen, J. E. Tierney. Simulation of Eocene extreme warmth and high climate sensitivity through cloud feedbacks. *Sci. Adv.*, **5**(9), 1–11 (2019). https://doi.org/10.1126/sciadv.aax1874
35. Zhu, C. J. Poulsen . Last Glacial Maximum (LGM) climate forcing and ocean dynamical feedback and their implications for estimating climate sensitivity. *Clim. Past*, **17**(1), 253–267, (2021). https://doi.org/10.5194/cp-17-253-2021
36. A. Goldner, N. Herold, M. Huber, M. The challenge of simulating the warmth of the mid-Miocene climatic optimum in CESM1. *Clim. Past*, **10**(2), 523–536, (2014) https://doi.org/10.5194/cp-10-523-2014
37. S. V. Nelson, Chimpanzee fauna isotopes provide new interpretations of fossil ape and hominin ecologies. *Proc. Royal. Soc. B* **280** (1773), 20132324 (2013).
